# CD169^+^ and HLA-DR^+^ extracellular vesicles are highly represented in human plasma and dynamically expressed in SARS-CoV-2 infection and long COVID-associated sequelae

**DOI:** 10.3389/fcimb.2026.1686186

**Published:** 2026-02-11

**Authors:** Marialaura Fanelli, Vita Petrone, Rossella Chirico, Luigi Coppola, Chiara Sorace, Chiara Cipriani, Giovanni Longo, Marco Girasole, Federica Collacchi, Claudia M. Radu, Martino Tony Miele, Alexandre Lucas, Elisabetta Teti, Vincenzo Malagnino, Marco Iannetta, Fabrice Malergue, Sergio Bernardini, Emanuela Balestrieri, Loredana Sarmati, Sandro Grelli, Antonella Minutolo, Claudia Matteucci

**Affiliations:** 1 Department of Experimental Medicine, University of Rome Tor Vergata, Rome, Italy; 2 Infectious Diseases Clinic, Policlinic of Tor Vergata, Rome, Italy; 3 Istituto di Struttura della Materia ISM - CNR, Rome, Italy; 4 Thrombotic and Hemorrhagic Diseases Unit Department of Medicine – DIMED University of Padua, Padua, Italy; 5 We-Met platform, Institut des Maladies Métaboliques et Cardiovasculaires (I2MC), plateau We-Met, Inserm UMR1297 and Université Paul Sabatier, Toulouse, France; 6 Department of Systems Medicine, University of Rome Tor Vergata, Rome, Italy; 7 Global Research Organization, Beckman Coulter Life Sciences, Marseille, France; 8 Virology Unit, Policlinic of Tor Vergata, Rome, Italy

**Keywords:** cell-to-cell communication, extracellular vesicles, flow cytometry, immune dysfunction, long COVID, myeloid activation markers, SARS-CoV-2

## Abstract

**Introduction:**

Elevated inflammation and immune dysregulation are the main consequences of severe acute respiratory syndrome coronavirus 2 (SARS-CoV-2) infection. The dysregulated inflammatory state persists after coronavirus disease 2019 (COVID-19), establishing the post-acute sequelae of SARS-CoV-2 infection in individuals with long COVID (LC). The role of CD169^+^ monocytes in the early diagnosis of SARS-CoV-2 infection and their association with severe outcomes were demonstrated in COVID-19 patients (COV). We aimed to delineate specific myeloid activation that characterizes the acute and post-acute phases of SARS-CoV-2 infection, evaluating the correlation between cellular and extracellular vesicles (EVs).

**Methods:**

Blood samples from COV, LC, and healthy donors (HD) were collected at Tor Vergata University Hospital in Rome. Plasmatic EVs were isolated by differential centrifugation and evaluated by flow cytometry and atomic force microscopy (AFM). Leukocyte subpopulations and different sizes of circulating EVs (100–200, 240–500, >500 nm) were characterized for HLA-DR and CD169 expression in COV, LC, and HD through flow cytometry. Serum inflammatory markers were assessed by the ELLA immunoassay system. The analyzed markers were associated with clinical and biochemical parameters in COV and LC.

**Results:**

The analysis of HLA-DR^+^, CD169^+^, and HLA-DR^+^CD169^+^ leukocytes confirmed our previous results in which the activated monocytes CD169^+^HLA-DR^+^ were found significantly high in COV, persisting in LC, and correlated differently with coagulation markers and inflammatory cytokines. Similar to cellular levels, the percentage and number of HLA-DR^+^CD169^+^ EVs were significantly elevated in COV and persisted in LC compared to HD. Different HLA-DR and CD169 expressions were found according to EV size in COV, LC, and HD, and correlations with biochemical parameters and circulating inflammatory markers were found. A positive correlation of HLA-DR and CD169 expression among monocytes and circulating EVs was found, supporting a possible connection between the two compartments and circulating inflammatory mediators. Moreover, the characterization by flow cytometry of EV cell derivation and cytokine cargo revealed EVs as sensitive indicators of both acute and persistent immune perturbations, bridging viral antigen persistence with inflammatory signaling in long COVID.

**Conclusion:**

Myeloid activation markers and inflammatory cytokines are dynamically expressed between blood cells and circulating extracellular vesicles, underlining multilevel cell-to-cell communications, opening new possibilities to monitor COVID-19 and long COVID-associated sequelae.

## 1 Introduction

The initial and persistent immunological dysfunction generated by severe acute respiratory syndrome coronavirus 2 (SARS-CoV-2) infection is still under investigation. Coronavirus disease 2019 (COVID-19) is an infectious disease associated with high morbidity and mortality in recent years. Hyperactivation of the innate immune system and dysregulation of adaptive immunity are crucial in the clinical outcome, contributing to the exacerbation of the disease. Indeed, cytokine storm, multisystem inflammatory syndrome, and other immunological reactions are common complications (Cao, 2020). Several studies have shown a compromised immune system in COVID-19 patients (Lv et al., 2022) that, in many cases, failed to recover full function, leading to the long COVID syndrome (LC). Individuals with LC show varied manifestations and sometimes overlapping symptoms, such as persistent fatigue, chest and muscle pain, headaches, shortness of breath, anosmia, muscle weakness, fever, cognitive dysfunction (brain fog), tachycardia, intestinal disorders, and skin manifestations (Turner et al., 2023; Ortona and Malorni, 2022; Zhao et al., 2024). In addition, LC individuals have been shown to have significant immunological impairment, characterized by a persistent immune dysregulation, particularly involving altered T-cell populations (Bohmwald et al., 2024; Minutolo et al., 2023). Moreover, the immune dysfunction found in LC individuals was characterized by a low-grade inflammation. Indeed, the persistence of pro-inflammatory cytokines at the serum level, such as Galectin-9, IL-6, CXCL10, CD163, and CCL2 (Berentschot et al., 2023), interleukin-6, interleukin-1β, and tumor necrosis factor-α (TNF-α), has been demonstrated (Ganesh et al., 2024; Low et al., 2023). It was also observed that several factors, such as gender, age, comorbidities, different SARS-CoV-2 variants and degree of acute infection, and genetic factors increase the risk of developing LC. In this context, it is necessary to investigate biomarkers, whose function is well-established in the context of the immune response and viral infections, in different cell and extracellular compartments to develop new strategies to characterize and monitor LC.

The major histocompatibility complex (MHC) II cell surface receptor (HLA-DR) is one of the main cell surface molecules expressed on antigen-presenting cells (monocytes, macrophages, and dendritic cells), B cells, and activated T lymphocytes and is responsible for antigen presentation to T cells and initiating the inflammatory cascade during infection (Nader, 2012; Mosaad, 2015). Studies have explored the role of HLA-DR in impairing innate immune responses in viral infections, evidencing downregulated expression on infected macrophages during human cytomegalovirus (HCMV) infection (Sandhu and Buchkovich, 2020) and on monocytes during syncytial virus (RSV) infection (Ahout et al., 2016). Downregulation of HLA-DR leads to a lower ability to control the activation and counteractivation of immune responses. Another protein, CD169 or Siglec-1, has increasingly captured the attention of the scientific community due to its expression on specific macrophage subpopulations of lymphoid tissue (Herzog et al., 2022). It is involved in the early stages of viral infections, in tumors, and in autoimmune diseases, increasing its importance in understanding immune responses and disease pathogenesis. Particularly, CD169^+^ macrophages, which are crucial for pathogen capture and antigen presentation, induce T- and B-cell responses and contribute to the amplification of immune reactions. CD169^+^ macrophages also secrete type I interferons (IFN-I) that mediate antiviral activity, but prolonged expression of IFN-I can lead to CD8^+^ T-cell depletion (Shaabani et al., 2016). The impairment of the immune response due to altered CD169 expression on macrophages has been shown in several viral infections, including RSV and HIV (Jans et al., 2018; Iannacone et al., 2010; Akiyama et al., 2017), and during the COVID-19 pandemic, the role of CD169 as a marker in the early diagnosis of SARS-CoV-2 infection and its association with severity and clinical outcome was demonstrated (Bourgoin et al., 2021; Bedin et al., 2021).

In addition to cellular receptors and release molecules, such as cytokines, that play a crucial and conspicuous role in the modulation of the immunological response, other factors being studied that represent a fundamental player in cellular communication are the extracellular vesicles (EVs). EVs are cell-derived membrane particles secreted into the extracellular space by cells, and they transfer genetic information (Théry et al., 2018; Yáez-Mó et al., 2015) in various physiological functions and pathological processes (Neven et al., 2017; Negahdaripour et al., 2022; Tassoni et al., 2025). Studies described that the release of EVs after viral infections could stimulate the immune system against pathogens, transporting viral protein (Booth et al., 2006; Keryer-Bibens et al., 2006), activating a robust T-cell response (Utsugi-Kobukai et al., 2003; Luketic et al., 2007; Walker et al., 2009; Giri and Schorey, 2008; Plazolles et al., 2011), inflammation (Caobi et al., 2020), and cell cycle deregulation (Nudel et al., 2015; Schorey and Harding, 2016). EVs have been proposed as the predominant exit pathway exploited by SARS-CoV-2 particles from invaginated regions (Eymieux et al., 2021) and in coronavirus fusion events (Sbarigia et al., 2022a). EVs can act positively or negatively toward SARS-CoV-2 infection, depending on their load, contributing not only to the spread of the virus but also to modulating target cell immune responses and susceptibility to infection (Sbarigia et al., 2022; Kumar et al., 2020; Xia et al., 2023; Serretiello et al., 2023; Choi et al., 2022). EVs have been studied in COVID-19 for their potential role as mediators of infection and associated pathological disorders (Pesce et al., 2022) and severity (Barberis et al., 2021; Bautista-Vargas et al., 2020; Rosell et al., 2021), but also activating a specific B-cell response, blocking virus entry (Troyer et al., 2021), or suppressing viral replication (Park et al., 2020; Sengupta et al., 2020). This evidence supports the dual role of EVs during SARS-CoV-2 infection, which can act both as a vehicle in the promotion of viral spread and as an element that can counteract the viral mechanisms.

The complex scenario of COVID-19 has required the discovery of new biomarkers capable of characterizing the disease as well as of predicting the long-term effects currently found in long COVID syndrome. Since the immune system plays a key role both in acute and post-infection phases, and EVs could reflect the activation state of the cell, a multilevel study could elucidate the pathophysiological combined functions. In this work, we aimed to delineate the specific myeloid activation that characterizes both the acute and post-acute phases of SARS-CoV-2 infection, with particular attention to the correlation between cellular and extracellular vesicles.

## 2 Materials and methods

### 2.1 Patient and healthy donor enrolment

Forty-eight (*n* = 48) SARS-CoV-2-positive (COV) patients were enrolled in an open-label study, promoted by the Departments of System Medicine and Experimental Medicine of the University of Rome, “Tor Vergata,” in the Infectious Diseases Clinic of Policlinico “Tor Vergata” (PTV). SARS-CoV-2 infection was assessed by the Allplex™ 2019-nCoV multiplex real-time polymerase chain reaction (PCR) assay, according to the manufacturer’s instructions.

Twenty-five (*n* = 25) long COVID individuals (LC) affected by post-acute sequelae of SARS-CoV-2 infection (PASC) were evaluated at the ambulatory of the Infectious Diseases Clinic of Policlinico “Tor Vergata” (PTV) starting from 3 months after the acute phase of infection. Ethical approval for the collection and use of human samples was obtained from the Ethics Committee of ‘Fondazione Tor Vergata’, COrona VIrus Disease: Safety and efficacy of experimental treatment (COVID_SEET prot.7562/2020, 9 April 2020, experimental register 46.20).

The COV and LC groups were divided according to the COVID-19 waves found from March 2020 to February 2022, based on recruitment. Five COVID-19 waves were identified, according to the literature: second wave (II) (September 2020–January 2021), third wave (III) (February 2021–June 2021), fourth wave (IV) (July 2021–September 2021), fifth wave (V) (October 2021–February 2022), and sixth wave (VI) (from March 2022). The clinical and biochemical data from COV and LC were collected and reported in **Tables 1**–**3**.

**Table 1:** Clinical and demographic characteristics of COVID-19 (COV) patients and Long COVID (LC) individuals.

Groups	COV N = 48		LC N = 25	
SEX F/M	Number	Percentage (%)	Number	Percentage (%)
	11/37	23/77	10/15	40/60
Age (mean ± SD)	59±13.20		54±16.33	
Severity (Acute phase)				
Moderate	37	77.08	20	80
Severe	10	20.83	5	20
N/A	1	2.08	0	0
Treatments (antiviral, corticosteroids, monoclonal)				
Yes	22	45.83	20	80
No	26	54.16	5	20
Vaccination				
No	46	95.83	8	32
Before	2	4.16	8	32
After	–		9	36
COVID-19 waves				
II (September 2020-January 2021)	43	89.58	6	24
III (February 2021-June 2021)	3	6.25	3	12
IV (July 2021-September 2021)	–	–	5	20
V (October 2021-February 2022)	2	4.16	11	44
Comorbidities				
Cardiovascular Yes	24	50	2	8
No	16	33.33	23	92
N/A	8	16.66	–	–
Diabetes Yes	12	25	4	16
No	27	56.25	21	84
N/A	9	18.75	–	–
Obesity Yes	9	18.75	5	20
No	31	64.58	20	80
N/A	8	16.66	–	–
Tumor Yes	5	10.41	-	-
No	35	72.91	25	100
N/A	–	–	–	–
	Interquartile Range (50) (25-75)		Interquartile Range (50) (25-75)	
Days of hospitalization during Acute Infection	2.00	1.00-3.00	6.00	5.00-16.00
Weeks after the Acute Infection	–	–	17.00	15.00-31.50

Clinical, demographic, and epidemiological features of patients with acute COVID-19 (COV, n = 48) and individuals with Long COVID (LC, n = 25). Data include sex distribution, age, disease severity during the acute phase, treatment received (antivirals, corticosteroids, monoclonal antibodies), vaccination status, and distribution across COVID-19 waves in Italy. Information on comorbidities such as cardiovascular diseases, diabetes, obesity, and tumors is also provided. Hospitalization duration during acute infection is expressed as median and interquartile range (IQR). For LC patients, the number of weeks after acute infection at the time of follow-up is reported. Values are presented as absolute numbers and percentages unless otherwise specified. Age is expressed as mean ± standard deviation (SD). Interquartile ranges (25th–75th percentile) are shown for continuous variables.

**Table 2:** Distribution of persistent symptoms among long COVID (LC, *n* = 25) patients at the time of follow-up.

LC symptoms		LC (*n* = 25)	
		Number	Percentage (%)
		10/15	40/60
Systemic	Yes	17	68
	No	6	24
	N/A	2	8
Cardiorespiratory	Yes	12	48
	No	11	44
	N/A	2	8
Cutaneous	Yes	7	28
	No	16	64
	N/A	2	8
Gastrointestinal	Yes	3	12
	No	21	84
	N/A	2	8
Neurologic	Yes	14	56
	No	9	36
	N/A	2	8
Psychiatric	Yes	8	32
	No	15	60
	N/A	2	8

Symptoms are categorized into six clinical domains: systemic (e.g., fatigue, malaise), cardiorespiratory (e.g., chest pain, dyspnea), cutaneous (e.g., skin rash), gastrointestinal (e.g., nausea, diarrhea), neurologic (e.g., brain fog, headache), and psychiatric (e.g., anxiety, depression). Values are presented as absolute numbers and percentages. “N/A” indicates missing or non-reported data. These data highlight the multisystemic nature of post-acute sequelae in SARS-CoV-2-infected individuals.

**Table 3:** Laboratory biomarkers, coagulation parameters, inflammatory indices, and comorbidity scores in patients with acute COVID-19 (COV, *n* = 48) and long COVID (LC, *n* = 25).

Biochemical data	Range values	COV		LC	
		Interquartile range		Interquartile range	
		(50)	(25–75)	(50)	(25–75)
Hematology					
Red blood cells	4.40–6.00 (10^6^/μL)	4.50	(4.08–5.13)	4.83	(4.54–5.25)
Hemoglobin	13–18 g/dL	13.40**	(11.80–14.40)	14.50	(13.08–15.68)
Hematocrit	36–51 (%)	39.70**	(35.60–42.60)	42.75	(39.80–46.08)
Platelets	150–450 (10³/μL)	206.00**	(166.00–240.00)	251.00	(216.75–302.50)
White blood cells	4.30–10.80 (10^5^/μL)	5.59**	(4.41–7.27)	7.35	(6.12–8.37)
Neutrophils	Abs count 10³/μL	4.09	(2.14–5.77)	4.48	(3.49–5.19)
	40–75 (%)	68.10**	(57.90–83.70)	61.20	(54.30–66.98)
Lymphocytes	Abs count 10³/μL	1.13***	(0.69–1.48)	2.03	(1.81–2.51)
	20–45 (%)	22.50*	(9.10–31.80)	28.90	(23.65–35.68)
Monocytes	Abs count 10³/μL	0.41	(0.32–0.59)	0.48	(0.42–0.57)
	3.4–11 (%)	7.20	(5.90–9.50)	6.95	(5.63–7.65)
Eosinophils	Abs count 10³/μL	0.01***	(0.00–0.02)	0.16	(0.06–0.22)
	0–7 (%)	0.10***	(0.00–0.60)	2.15	(0.98–3.70)
Basophils	Abs count 10³/μL	0.01***	(0.01–0.03)	0.04	(0.03–0.05)
	0–1.5 (%)	0.30***	(0.20–0.40)	0.50	(0.40–0.70)
Coagulation					
PT %	70–130 (%)	86.50***	(84.25–93.00)	108.00	(96.00–115.00)
PT-INR	0.80–1.20	1.12***	(1.07–1.13)	0.95	(0.92–1.03)
PT sec	Sec	13.30***	(12.40–14.20)	11.40	(10.90–12.00)
aPTT ratio	0.80–1.20	1.01*	(0.92–1.07)	1.08	(0.99–1.18)
aPTT sec	25–38.50 (sec)	28.65*	(25.90–30.58)	30.70	(28.30–33.70)
Fibrinogen	200–400 (mg/dL)	516.00***	(418.00–589.00)	301.00	(256.25–368.25)
D-dimer	0–500 (ng/mL)	571.00***	(449.50–990.50)	273.00	(190.00–382.75)
Antithrombin III	75–128 (%)	105.00	(95.00–118.00)	–	–
Clinical chemistry					
Azotemia	18–55 (mg/dL)	43.00**	(27.25–67.50)	30.00	(22.00–36.50)
Potassium	3.50–5.10 (mEq/L)	4.10	(3.80–4.60)	4.30	(4.00–4.40)
Albumin	3.20–4.60 (gr/dL)	3.54*	(3.13–4.09)	–	–
AST	5–34 (U/L)	38.00***	(25.00–60.50)	26.00	(20.00–30.50)
ALT	0–55 (U/L)	31.00	(20.75–67.50)	29.00	(19.00–38.00)
LDH	125–220 (U/L)	336.00***	(235.00–436.00)	192.00	(164.00–215.00)
Amylase	20–160 (U/L)	63.00	(48.00–100.00)	–	–
Lipase	<59 (U/L)	41.50	(23.75–98.25)	33.00	(26.00–38.00)
CRP	0–5 (mg/L)	38.00***	(12.50–95.00)	2.00	(1.00–4.00)
Inflammatory and infection indices					
SII	424.06 10³/μL	**620.86**	(331.00–1,592.63)	**491.81**	(337.56–701.13)
NLR	1.94	2.82**	(1.88–2.82)	**2.02**	(1.49–2.79)
PLR	127.30	169.23**	(125.30–270.47)	126.27	(97.56–161.78)
CD169 RMFI	<3	22.82***	(10.88–39.49)	1.67	(1.36–2.28)
Charlson comorbidity index					
		3	(2–4.25)	1	(0–2)
		Number	Percentage (%)	Number	Percentage (%)
	0	6	12.50	8	32
	1	3	6.25	6	24
	2	6	12.50	6	24
	3	11	22.91	2	8
	4	6	12.50	1	4
	5	4	8.33	2	8
	6	2	4.16	–	–
	7	1	2.08	–	–
	8	2	4.16	–	–
	9	–	–	–	–
	10	1	2.08	–	–
	N/A	6	12.50	–	–

Data are presented as interquartile ranges (median, 25th–75th percentiles), with reference ranges included for each variable where applicable. The dataset includes hematological values (e.g., RBC, hemoglobin, WBC, differential counts), coagulation parameters (e.g., PT, INR, fibrinogen, D-dimer), clinical chemistry values (e.g., AST, ALT, LDH, CRP), and calculated inflammatory indices such as the neutrophil-to-lymphocyte ratio (NLR), platelet-to-lymphocyte ratio (PLR), systemic immune-inflammation index (SII), and CD169 relative median fluorescence intensity (RMFI). The Charlson comorbidity index (CCI) distribution is also reported. Asterisks indicate statistically significant differences between the COV and LC groups (*p < 0.05, **p < 0.01, ***p < 0.001). These data highlight sustained biochemical and inflammatory alterations during the acute phase of COVID-19 compared to the post-acute (LC) phase, suggesting resolution of systemic inflammation in most LC.

Twenty-two (*n* = 22) HD, who were not infected with SARS-CoV-2, were obtained from people attending the local blood transfusion center and were sent to the Virology Unit for screening. The donors have been matched for age and sex to the best extent possible with the patients and have provided written informed consent.

### 2.2 Plasmatic extracellular vesicle isolation

Plasma samples were obtained from whole blood, collected in spray-coated K2EDTA with BD Vacutainer^®^, by centrifugation for 10 min at 1,000–2,000×*g* at 24°C, and stored at −80°C until use. In accordance with the guidelines proposed by MISEV 2024 (Welsh et al., 2024), the first 2 mL of whole blood was not used to obtain plasma, and the sample was processed quickly to prevent platelet activation. For EV isolation, 300 μL of COV, LC, and HD plasma were used. For the isolation of EVs, the MiniSpin^®^ centrifuge (Eppendorf) was used (rotor 7 cm, minor radius 3.35 cm, and larger radius 2.8 cm). An initial centrifugation was performed at 13,000 rpm (13,226×*g*) for 5 min at 24°C. The supernatant was recovered and diluted with 200 μL of Dulbecco’s phosphate-buffered saline (PBS) filtered with a 0.22-μm filter. Subsequent centrifugations were performed at the same speed. In the final step, the supernatant was removed, and EV pellets were recovered and resuspended in 300 μL of PBS. The EV aliquots obtained were stored at −80°C until use. The centrifuge, temperature, and time information were used to calculate the k-factor (*K*) and sedimentation coefficient (*S*) of the recovered particles.


$$k=2.533\text{ x }10^{5}\text{x }\ln(\frac{rmax}{rmin}){\left(\frac{RPM}{1000}\right)}^{2}$$

$$s=\frac{k}{Time\;of\;centrifugation}$$

The calculation of the sedimentation coefficient (*S*) showed a value of 53.65. According to MISEV, particles with *S* coefficients in the range of 15–150 are recovered from the “larger EV” centrifugation conditions.

### 2.3 Characterization of EVs by atomic force microscopy

We characterized the HD, COV, and LC EVs using atomic force microscopy (AFM). We have used the Park NX-12 (Park Systems Inc., Korea) AFM, which is mounted on an Olympus IX inverted optical microscope equipped with a high-resolution camera. We used commercial AFM cantilevers, namely, Bruker DNP-10, choosing the sensor with a nominal elastic constant of 0.12 N/m. Before all experiments, the sensors were calibrated using the built thermal noise routines to determine the resonant frequency and the corresponding mechanical properties of the sensor (Hutter and Bechhoefer, 1993). The measurements were performed in air using a non-contact modality, with an oscillation of 10 nm and a 10% oscillation reduction as the threshold. In this modality, the tip is placed in oscillation over the sample, and the tip–sample interaction produces alterations in the oscillation which are used as feedback to reconstruct the morphology of the sample. In this modality, the tip–sample interaction is minimal, ensuring that the measurement does not alter the size and shape of the EVs (**Supplementary Material Figures S1A, B**) (Sbarigia et al., 2022b).

### 2.4 Analysis of CD169 and HLA-DR expression in blood cells by flow cytometry

Blood cells (30 μL) from the COV, LC, and HD were vortexed for 5 min and incubated for 15 min in the dark at room temperature with 1.5 mL of VersaLyse Lysing Solution (Beckman Coulter, BC) to lyse red blood cells and select a total leukocyte population. Antibodies of interest were incubated for 15 min in the dark on ice. A total of 10 μL of IOTest myeloid activation antibody cocktail composed of anti-CD169-phycoerythrin (PE) (clone 7–239), anti-CD64-Pacific Blue (PB), and HLA-DR (APC) (clone 22) (Beckman Coulter) was used.

Specifically, we used the monoclonal antibody Immu357 (A74781, liquid—0.5 mL), which recognizes an epitope carried on a monomorphic 29–33-kDa protein identified as HLA-DR. This clone targets a conserved determinant shared by HLA-DR molecules and does not discriminate between different HLA-DRB1 alleles.

The stained cells were analyzed via CytoFLEX (Beckman Coulter) and CytExpert 2.3 software (BC). CD169 expression was represented as the ratio of CD169 median fluorescence intensity (MFI) between HLA-DR-positive monocytes and lymphocytes (RMFI), and the percentage of positive cells for HLA-DR and CD169 markers was also analyzed as described in previous studies (Minutolo et al., 2021; Fanelli et al., 2024). The cells (leukocytes, lymphocytes, monocytes, granulocytes), CD169^+^, HLA-DR^+^, and CD169^+^HLA-DR^+^ gating strategy were reported in **Supplementary Figure S2** in the **Supplementary Material**.

### 2.5 Analysis of CD169 and HLA-DR expression in plasmatic EVs by flow cytometry

EVs, previously isolated from the plasma samples of the COV, LC, and HD, were characterized by flow cytometry for dimensions and phenotypic characteristics. The new generation flow cytometer (CytoFLEX, Beckman Coulter) equipped with 3 lasers (405, 488, and 638 nm) and 13 band pass filters (450/45, 525/40, 585/42, 610/20, 660/10, 690/50, 712/25, and 780/60 and capable of detecting sizes up to 100 nm) was used to detect EVs. FSC and SSC were resulting from a 488-nm laser line excitation, while vSSC (violet SSC) was resulting from a 405-nm laser line excitation.

Flow cytometry was used to identify the EV subtypes according to size, as suggested by the guidelines on minimal information for the study of extracellular vesicles (MISEV 2024) and as reported in a recent study (“Increased circulating CD62E^+^ endothelial extracellular vesicles predict severity and in-hospital mortality of COVID-19 patients”).

For the analysis of EVs, a gating strategy was generated by using a combination of FITC-labeled fluorescent Megamix-Plus SSC (Cat# 7803, Biocytex, France) and Megamix-Plus FSC beads (Cat# 7802, Biocytex, France), hereby termed as Gigamix. The Gigamix contains beads of sizes 0.1, 0.16, 0.2, 0.24, 0.3, 0.5, and 0.9 μm. Acquisition settings for EVs were adjusted as FSC gain at 170, SSC gain 100, and vSSC gain at 500. The gating strategy is reported in the **Supplementary Material, Figure S3**.

Before the acquisition of EVs, the flow cytometer was cleaned with a cleaning buffer, and the filtered PBS was read before reading the sample. Controls included for all EV analyses consisted of a negative control with filtered PBS (**Supplementary Figure S4**) and unstained samples.

For phenotypic characterization, the EVs (20 μL) were incubated for 20 min at room temperature in the dark with 3 μL of IOTest myeloid activation antibody cocktail composed of anti-CD169-phycoerythrin (PE) (clone 7–239), anti-CD64-Pacific Blue (PB), and HLA-DR (APC) (clone 22) (Beckman Coulter). The stained EVs were then washed with filtered 0.22 μm filter PBS, centrifuged at 13,000 rpm × 60 s, and resuspended in 50 μL of filtered PBS for acquisition. The concentration and quantification of EVs (number of events/µL, total number of events, and percentage) were calculated using CytoFLEX (Beckman Coulter, USA) and CytExpert 2.3 software (Beckman Coulter, USA), and 150,000 events were acquired in the 0.1- and 0.9-μm range.

### 2.6 Statistical analysis

Statistical analysis of groupwise expression levels was performed using the non-parametric Mann–Whitney test for comparisons between two independent samples, or the Kruskal–Wallis test followed by Bonferroni’s correction for multiple comparisons when more than two groups were evaluated. Pairwise associations between continuous variables were assessed using the Spearman correlation coefficient. To account for multiple testing in correlation analyses, *p*-values from Spearman tests were adjusted using the Benjamini–Hochberg false discovery rate (FDR) method, and significance was set at FDR-adjusted *p*-value (*q*-value) <0.05. All statistical analyses were conducted using SPSS statistical software (version 23.0 for Windows, USA).

## 3 Results

### 3.1 Altered expression of CD169 and HLA-DR in leukocyte subpopulations in COVID-19 patients and long COVID individuals

**Figure 1:**
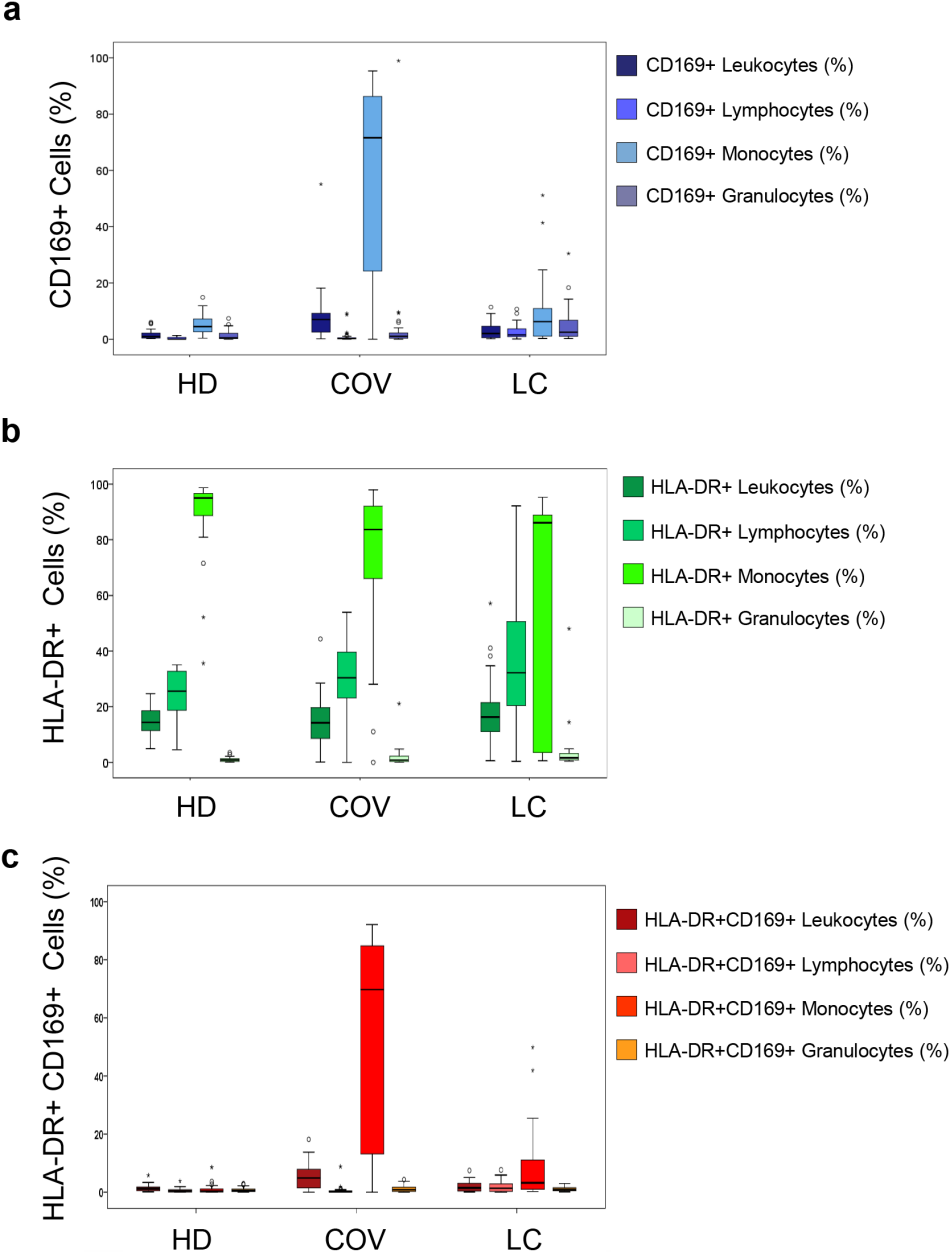
Expression of CD169 and HLA-DR in leukocyte subpopulations of healthy donors (HD), COVID-19 patients (COV), and individuals with long COVID (LC). Box plots show the percentages of cells positive for CD169 and HLA-DR and double-positive CD169^+^HLA-DR^+^ markers across different leukocyte subsets, including total leukocytes, lymphocytes, monocytes, and granulocytes. Each box represents the interquartile range (IQR), with the horizontal line indicating the median; whiskers denote the full data range, and outliers are shown as individual points. **(a)** CD169^+^ expression was markedly increased in monocytes and total leukocytes in COV patients and remained elevated in granulocytes and lymphocytes in LC individuals. **(b)** HLA-DR expression was significantly elevated in monocytes in both COV and LC compared to HD and in granulocytes from LC individuals. **(c)** The proportion of double-positive HLA-DR^+^CD169^+^ monocytes and lymphocytes was significantly higher in both COV and LC compared to HD. Statistical comparisons were performed using the Mann–Whitney U test. *p < 0.05, *p* < 0.01, *p < 0.001.

The expression levels of CD169 and HLA-DR on different leukocyte subpopulations such as lymphocytes, monocytes, and granulocytes were evaluated in HD (*n* = 18), COV (*n* = 48), and LC (*n* = 25) by flow cytometry as reported in **Figure 1**. Results are expressed as percentages of positive cells in box plots by groups. The statistical comparison is reported in **Table 4**. Considering total leukocytes, CD169 expression was significantly upregulated in all leukocyte subsets in COV patients compared to HD and LC, with the highest induction observed in monocytes (*p* < 0.0001 vs. HD; *p* < 0.0001 vs. LC), reflecting the acute type I interferon activation during infection. In LC, all leukocyte subsets maintained a moderate elevation in CD169 expression compared to HD (*p* < 0.001), significantly lower than COV, demonstrating post-infection downregulation but also suggesting persistent interferon stimulation.

**Table 4:** Kruskall wallis statistical analysis of expression of CD169, HLA-DR in leukocyte subpopulations in COV patients and in LC individuals.

% of positive cells		COV vs HD	LC vs HD	COV vs LC
**CD169+**	Leukocytes	0.000	0.000	0.001
	Lymphocytes	0.139	0.000	0.000
	Monocytes	0.000	0.000	0.000
	Granulocytes	0.308	0.004	0.006
**HLA-DR+**	Leukocytes	0.737	0.601	0.327
	Lymphocytes	0.040	0.063	0.466
	Monocytes	0.000	0.000	0.230
	Granulocytes	0.658	0.019	0.015
**HLA-DR+ CD169+**	Leukocytes	0.001	0.000	0.001
	Lymphocytes	0.000	0.000	0.000
	Monocytes	0.000	0.000	0.000
	Granulocytes	0.285	0.301	0.570

Analyzing the HLA-DR expression, no significant differences were observed in total leukocytes among the three groups; however, lymphocytes were significantly elevated in both COV and LC compared to HD (*p* < 0.0001), indicating sustained activation. As previously demonstrated, a lower expression of HLA-DR was found in monocytes of COV and LC compared to HD (*p* < 0.0001). Interestingly, a slight but significant increase in HLA-DR expression on granulocytes was found in COV and LC compared to HD (*p* = 0.015; *p* = 0.019, respectively), suggesting a delayed or persistent granulocyte activation in LC.

Finally, double-positive HLA-DR^+^CD169^+^ monocytes were confirmed as significantly high in COV and lower as well as persisting in LC.

### 3.2 Differential expression of HLA-DR and CD169 on plasma EVs in COVID-19, long COVID, and healthy donors

**Figure 2:**
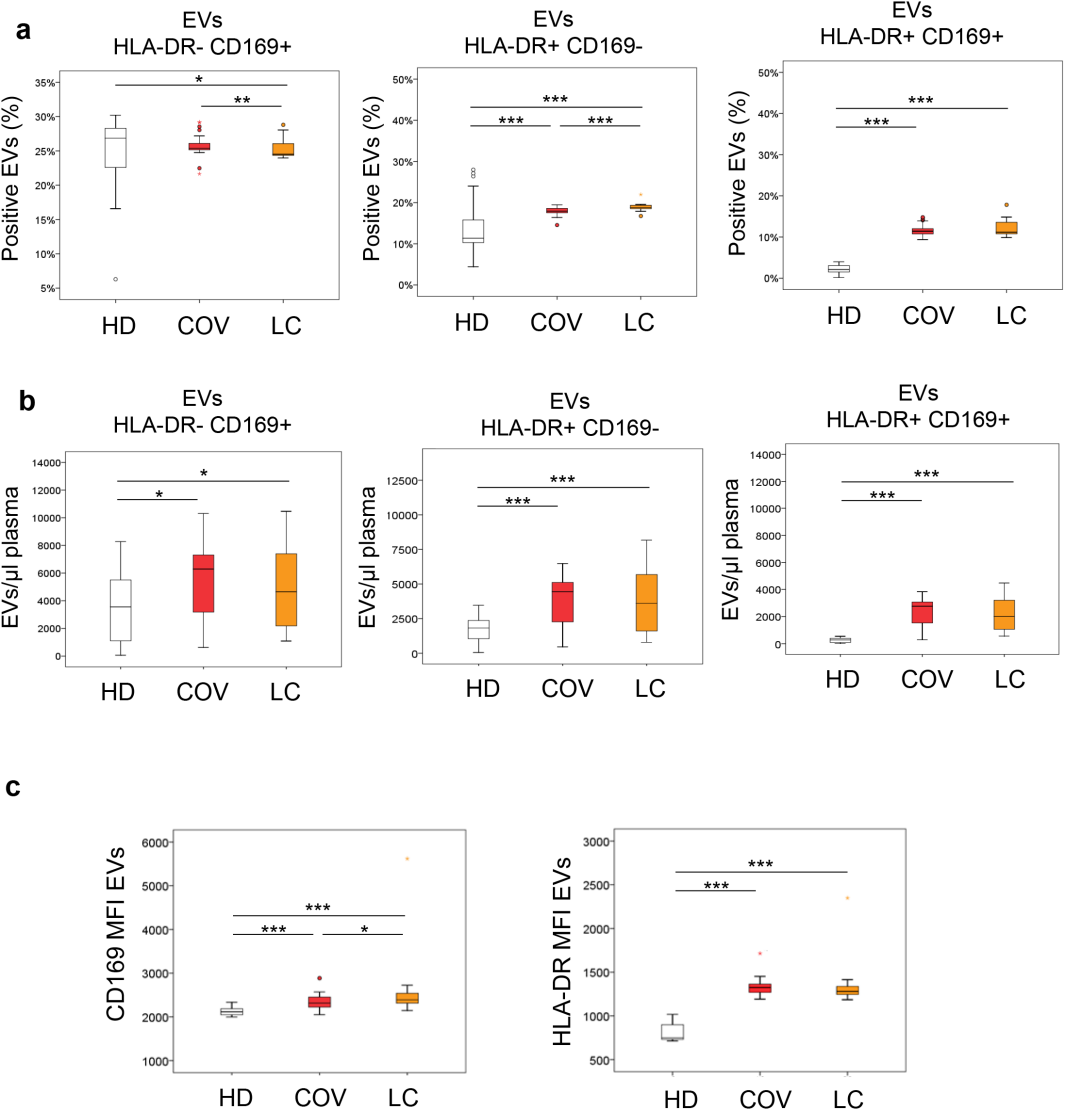
Characterization of plasma extracellular vesicles (EVs) expressing HLA-DR and CD169 in healthy donors (HD), COVID-19 patients (COV), and Long COVID patients (LC). EVs were isolated from plasma and analyzed by flow cytometry to assess the surface expression of HLA-DR and CD169. Panels show the percentage (%) of EVs (top row of each section) and absolute number of EVs per µL of plasma (bottom row of each section) for the following subsets: HLA-DR⁻CD169^+^**(a)**, HLA-DR^+^CD169^-^**(b)**, and HLA-DR^+^CD169^+^**(c)**. The last row displays the median fluorescence intensity (MFI) of CD169 and HLA-DR on EVs. Data are presented as box-and-whisker plots. *p < 0.05, **p < 0.01, ***p < 0.001; comparisons were performed using Kruskal–Wallis test followed by Dunn’s post hoc test for multiple comparisons.

To assess the involvement of plasmatic EVs expressing HLA-DR and CD169 in COV, LC, and HD, flow cytometry analysis, able to identify and characterize EVs, was performed as reported in the gating strategy described in the *Materials and Methods* (**Supplementary Figure S3**). The percentages and the number of EVs relative to the µL of plasma for the three different groups were determined (**Figure 2**). The percentage of HLA-DR**^−^**CD169^+^ EVs (**Figure 2A**) was significantly lower in LC with respect to HD (*p* = 0.015) and to COV (*p* = 0.002). No difference was observed between the HD and COV groups (*p* = 0.100). The percentage of HLA-DR^+^CD169^−^ EVs was significantly higher in COV and LC with respect to HD (*p* < 0.001; *p* < 0.001) and was lower in COV compared to LC (*p* < 0.001). As reported in **Figure 2A**, the percentage of HLA-DR^+^CD169^+^ EVs was significantly higher in COV and LC with respect to HD (*p* < 0.001; *p* < 0.001). No difference between COV and LC was observed (*p* = 0.898). The number of EVs expressing HLA-DR and CD169 for the µL of plasma was also evaluated (**Figure 2B**). Interestingly, the number of HLA-DR^−^CD169^+^, HLA-DR^+^CD169^−^, and HLA-DR^+^CD169^+^ EVs was significantly higher in COV and LC with respect to HD (*p* = 0.010; *p* = 0.047; *p* < 0.001; *p* < 0.001; *p* < 0.001; *p* < 0.001). No significant differences emerged between the COV and LC groups (*p* = 0.569; *p* = 0.981; *p* = 0.754). The MFI values of CD169^+^ and HLA-DR^+^ EVs were analyzed. As reported in **Figure 2C**, compared to HD, the CD169 MFI of EVs was significantly higher in COV and LC (*p* < 0.001; *p* < 0.001). The EV CD169 MFI was also higher and significant in LC with respect to COV (*p* = 0.034). In addition, in both COV and LC compared to HD, the EV HLA-DR MFI was found to be high (*p* < 0.001; *p* < 0.001). No significant differences were observed between COV and LC (*p* = 0.087).

### 3.3 Differential expression of EV subtypes carrying HLA-DR and CD169 markers in plasma of healthy donors, COVID-19 patients, and long COVID patients

**Figure 3:**
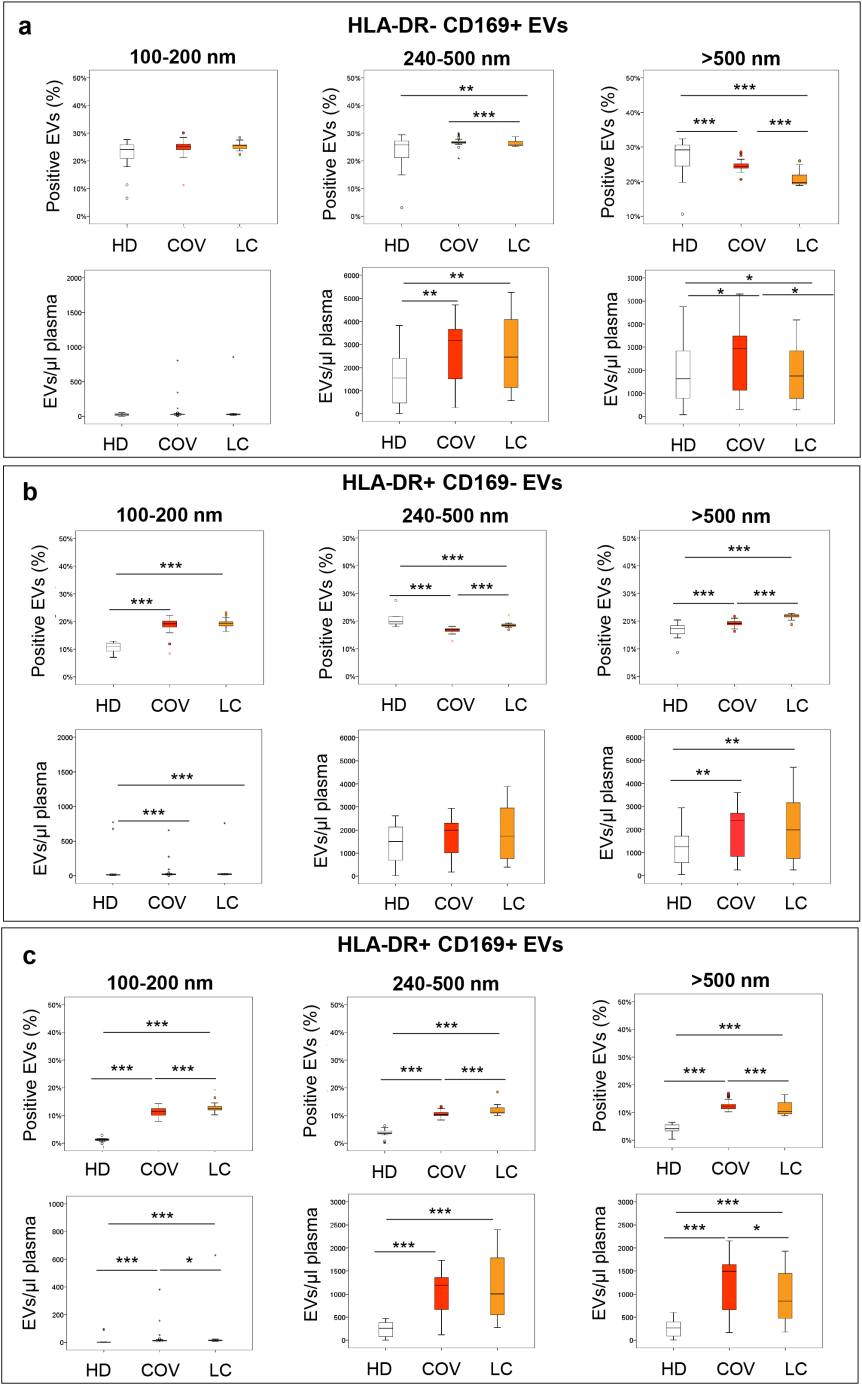
Flow cytometry analysis of different subpopulation plasmatic EVs reported as percentage and number of HLA-DR and CD169 positive in COV, LC, and HD. Representation of the CD169^+^, HLA-DR^+^, and CD169^+^HLA-DR^+^ EVs across the size ranges of 100–200, 240–500, and >500 nm analyzed in COV (red box), LC (orange box), and HD (white box). **(a)** Evaluation of the percentage and the number of different ranges of 100–200, 240–500, and >500 nm of plasmatic HLA-DR^−^CD169^+^ EVs (events/µL of plasma). **(b)** Evaluation of the percentage and the number of different ranges of 100–200, 240–500, and > 500 nm of plasmatic HLA-DR^+^CD169^−^ EVs (events/µL of plasma). **(c)** Evaluation of the percentage and the number of different ranges of 100–200, 240–500, and >500 nm of plasmatic HLA-DR^+^CD169^+^ EVs (events/µL of plasma). Plasmatic EVs were analyzed by flow cytometry using the gating strategy shown in the **Supplementary Material**. The non-parametric Kruskal–Wallis test and Bonferroni’s correction were used to compare the groups analyzed (***p ≤ 0.000, **p ≤ 0.001, *p ≤ 0.05).

Plasma-derived EVs were analyzed by flow cytometry for surface expression of HLA-DR and CD169 markers in the three study groups—HD, COV, and LC. **Figure 3** displays the box plots representing both the percentage of positive EVs and the absolute number of EVs per microliter of plasma for the subsets HLA-DR^−^CD169^+^, HLA-DR^+^CD169^−^, and HLA-DR^+^CD169^+^. Through flow cytometry and the gating strategy generated by the Gigamix assay, it was possible not only to identify the total EVs but also to divide them into three different groups according to their size. Three different groups of EVs were examined: 100–200 nm (small), 240–500 nm (medium), and larger than 500 nm (large), as shown by the gating strategy (**Supplementary Figure S3**). The EVs of different sizes expressing the markers CD169 and HLA-DR were evaluated.

The percentage and the number of small EVs (100–200 nm) HLA-DR^−^CD169^+^ showed no significant difference in the COV, LC, and HD groups (EV percentages: COV vs. HD: *p* = 0.054; LC vs. HD: *p* = 0.098; LC vs. COV: *p* = 0.667; EV numbers: COV vs. HD: *p* = 0.830; LC vs. HD: *p* = 0.526; LC vs. COV: *p* = 0.280) (**Figure 3A**).

On the other hand, larger EVs showed significant differences in the three groups analyzed. In particular, the analysis of HLA-DR^−^CD169^+^ EVs in the 240–500-nm size range showed a significantly higher percentage in COV with respect to LC (*p* = 0.001) and in LC than HD (*p* = 0.007). No significant difference was observed between COV and HD (*p* = 0.068). Considering the number of HLA-DR^−^CD169^+^ EVs/µL of plasma, no significant difference was found between COV and LC (*p* = 0.935), but significant differences were found between COV (*p* = 0.002) and LC (*p* = 0.009) compared to HD.

The HLA-DR^−^CD169^+^ EVs larger than 500 nm showed a significantly lower percentage in COV (*p* = 0.001) and LC (*p* < 0.001) than HD, but the COV group showed a significantly higher percentage than LC (*p* < 0.001). In contrast to the percentage, the number of HLA-DR^−^CD169^+^ EVs/µL of plasma larger than 500 nm was higher and statistically significant in the COV than in the LC and HD groups (*p* = 0.032; *p* = 0.024). Likewise, it was significantly higher in the LC group than in the HD (*p* = 0.025).

The expression in both the percentage and number of HLA-DR^+^CD169^−^ EVs of different sizes in the three groups analyzed was also evaluated as reported in **Figure 3B**. The percentage of HLA-DR^+^CD169^−^ EVs between 100 and 200 nm was significantly higher in COV (*p* < 0.001) and LC (*p* < 0.001) than in HD. A similar trend was observed analyzing the number of HLA-DR^+^CD169^−^ EVs (COV vs. HD: *p* = 0.001; LC vs. HD: *p* < 0.001; HD mean 12.41 EVs/µL of plasma; COV 21.60 EVs/µL of plasma; LC 24.50 EVs/µL of plasma). In both analyses, no significant differences between COV and LC emerged (percentages HLA-DR^+^CD169^−^ EVs COV vs. LC: *p* = 0.496; HLA-DR^+^CD169^−^ EVs/μL plasma COV vs. LC: *p* = 0.187).

The percentage of HLA-DR^+^CD169^−^ EVs in the 240–500-nm range was significantly lower in COV (*p* < 0.001) and LC (*p* < 0.001) than in HD and significantly higher in LC than in COV (*p* < 0.001). No significant evidence was observed regarding the number of HLA-DR^+^CD169^−^ EVs/µL of plasma in the 240–500-nm range (COV vs. HD: *p* = 0.176; LC vs. HD: *p* = 0.247; COV vs. LC: *p* = 0.553).

The analysis of HLA-DR^+^CD169^−^ EVs larger than 500 nm showed a high and significant percentage in COV (*p* = 0.001) and LC (*p* < 0.001) compared to HD; in particular, the LC group showed a significantly higher percentage compared to COV (*p* < 0.001).

The number of HLA-DR^+^CD169^−^ EVs/μL plasma in both COV (*p* = 0.003) and LC (*p* = 0.014) was significantly higher than in HD. No significant differences were observed between the number of HLA-DR^+^CD169^−^ EVs/μL plasma in COV and LC (*p* = 0.745).

Finally, the percentage and number of EVs positive for both HLA-DR and CD169 markers were analyzed considering the three size groups 100–200, 240–500, and 500 nm in COV, LC, and HD (**Figure 3C**). The percentage of HLA-DR^+^CD169^+^ EVs between 100 and 200 nm was significantly higher in COV (*p* < 0.001) and LC (*p* < 0.001) with respect to HD, while LC showed a significantly higher percentage than COV (*p* < 0.001). Similar results were found in the number of HLA-DR^+^CD169^+^ EVs (COV vs. HD: *p* < 0.001; LC vs. HD: *p* < 0.001; COV vs. LC: *p* = 0.026; HD mean 0.53 EVs/µL of plasma; COV 4.99 EVs/µL of plasma; LC 16.05 EVs/µL of plasma).

The percentage of HLA-DR^+^CD169^+^ EVs in the range of 240–500 nm was significantly higher in COV (*p* < 0.001) and LC (*p* < 0.001) than in HD, while LC showed a significantly higher percentage than COV (*p* = 0.001). When analyzing the number of HLA-DR^+^CD169^+^ EVs for μL of plasma, both COV and LC showed a significantly higher number with respect to HD (*p* < 0.001; *p* < 0.001). No difference between COV and LC was observed (*p* = 0.609).

The percentage and number of HLA-DR^+^CD169^+^ EVs with size greater than 500 nm were significantly higher in COV and LC than HD (percentages of EVs: *p* < 0.001; *p* < 0.001; number of EVs: *p* < 0.001; *p* < 0.001); notably, LC still showed significantly higher values than HD (percentage of EVs: *p* = 0.001; number of EVs: *p* = 0.034). These data suggest that EV subsets bearing HLA-DR and/or CD169 markers are significantly dysregulated in COVID-19 and long COVID patients compared to healthy individuals. In particular, LC patients show a distinct EV signature, with elevated CD169 expression and altered distribution of EV subpopulations, which may reflect persistent immune activation.

### 3.4 CD169 and HLA-DR-positive EVs correlate with CD169 RMFI and monocytes expressing CD169 and HLA-DR in COV and LC

**Figure 4:**
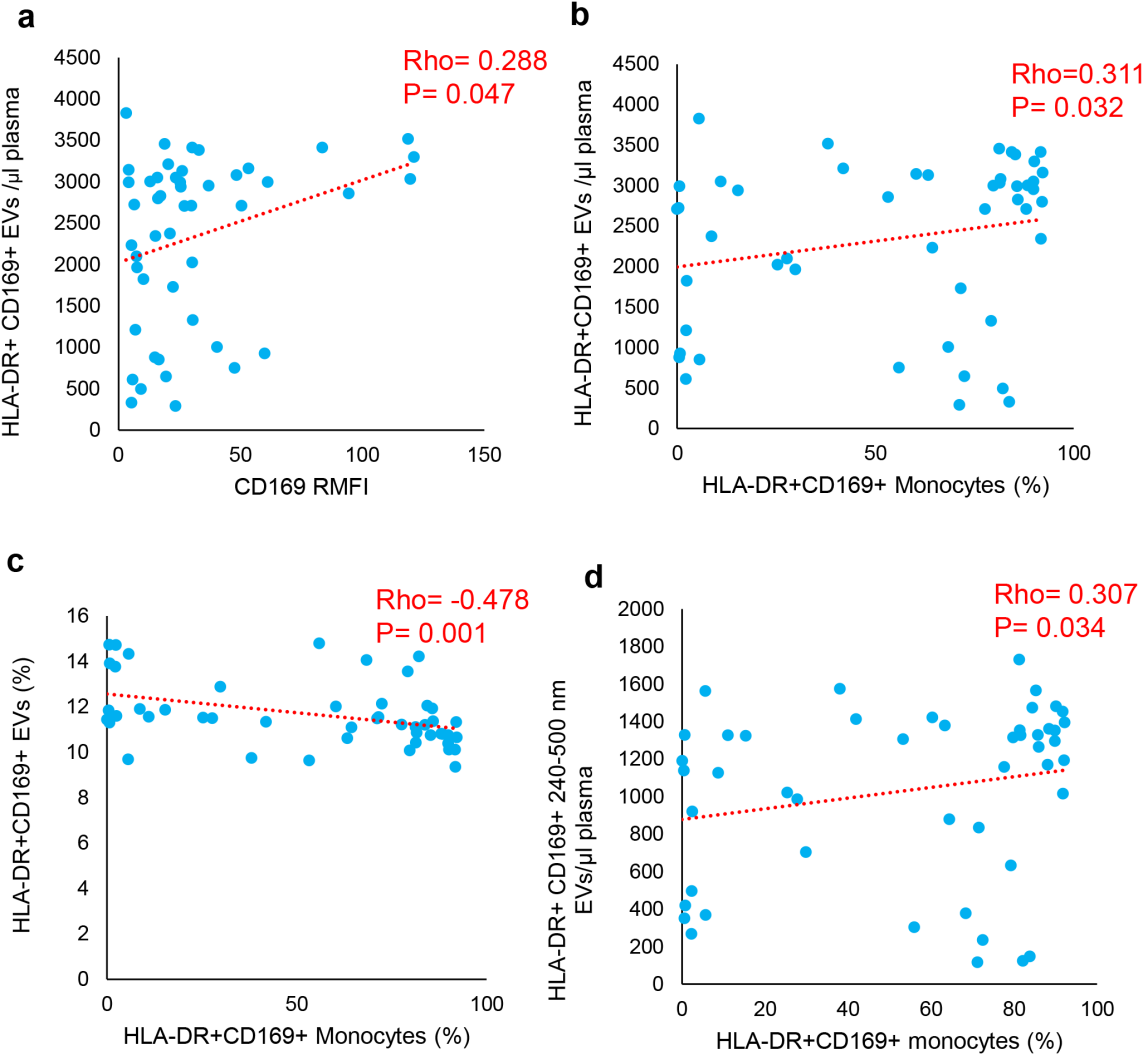
Correlations of CD169 and HLA-DR positive EVs with CD169 RMFI and monocytes expressing CD169 and HLA-DR in COV. The correlations represented in scatter plots were analyzed through Spearman correlation coefficient. The values of Spearman's Rho (Rho) and p value were reported. Direct correlation was observed between CD16 RMFI and HLA-DR+CD169+ EVs/μl plasma **(a)**; HLA-DR+CD169+ Monocytes vs EVs/μl plasma **(b)** and EVs percentage **(c)**, and between HLA-DR+CD169+ Monocytes vs EVs/μl plasma (240-500 nM, **(d)**)

**Figure 5:**
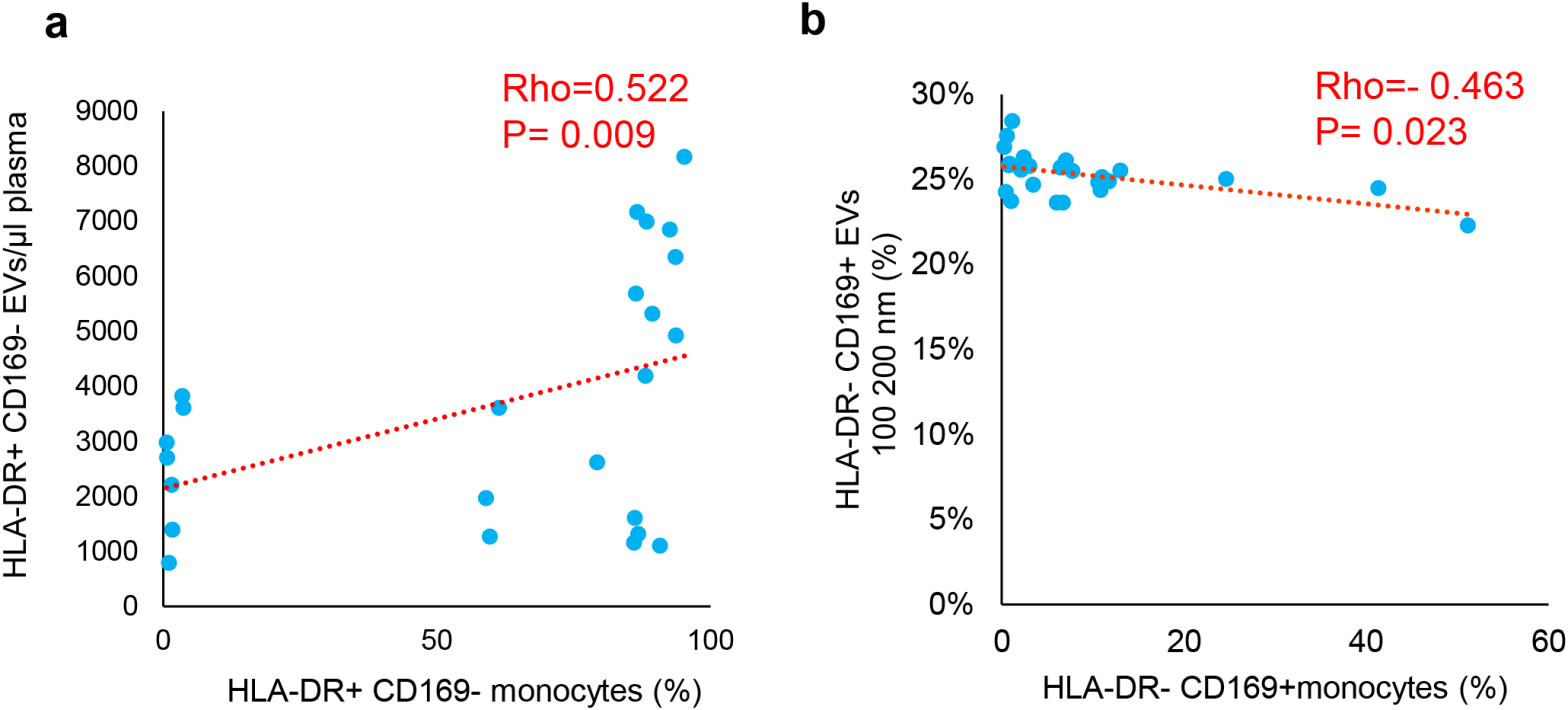
Correlations of CD169 and HLA-DR positive EVs with monocytes expressing CD169 and HLA-DR in LC. The correlations represented in scatter plots were analyzed through Spearman correlation coefficient. The values of Spearman's Rho (Rho) and p value were reported. Percentage of positive HLA-DR+CD169- Monocytes vs EVs/μl plasma **(a)**, and vs HLA-DR+CD169+ EVs 100-200 nm **(b)**.

The association between circulating HLA-DR^+^ and CD169^+^ EVs and cells expressing the same markers was tested both in COV (**Figure 4**) and LC through the Spearman correlation analysis (**Figure 5**). In COV, a direct correlation of the number of HLA-DR^+^CD169^+^ EVs with the CD169 RMFI (Rho = 0.288, *p* = 0.047) and with the percentages of HLA-DR^+^CD169^+^ monocytes (Rho = 0.311, *p* = 0.032) was observed. Moreover, a direct correlation between the number of HLA-DR^−^CD169^+^ EVs and the percentages of HLA-DR^−^CD169^+^ monocytes was shown (Rho = 0.374, *p* = 0.009). Inversely to the number of EVs, the percentage of HLA-DR^+^CD169^+^ EVs was indirectly correlated with the percentage of HLA-DR^+^CD169^+^ monocytes (Rho = −0.478, *p* = 0.001). Considering the analysis of EVs of different sizes, a direct correlation between the number of HLA-DR^+^CD169^+^ EVs in the range of 240–500 nm with the percentage of HLA-DR^+^CD169^+^ monocytes (Rho = 0.307, *p* = 0.034) was observed (**Figure 4**). In the LC group, a direct correlation of the number of HLA-DR^+^CD169^−^ EVs with the percentages of HLA-DR^+^CD169^−^ monocytes (Rho = 0.522, *p* = 0.009) was observed. There was an inverse correlation between the percentages of HLA-DR^−^CD169^+^ EVs in the range size 100–200 nm with the percentages of HLA-DR^−^CD169^+^ monocytes (Rho = −0.463, *p* = 0.023) (**Figure 5**).

### 3.5 Correlation of leukocyte subpopulations expressing CD169 and HLA-DR with biochemical data in COV and LC

The association between CD169 markers in leukocytes with biochemical data and inflammatory indices was tested through the Spearman correlation analysis (**Table 5**). In COVID-19 patients, only some biochemical parameters were found to be correlated with CD169 and HLA-DR markers, mainly negatively, such as aPTT ratio, azotemia, AST, ALT, and CRP.

**Table 5:** Spearman correlations between the percentage of immune cells positive for CD169, HLA-DR, or both (HLA-DR^+^CD169^+^) and clinical laboratory parameters in COVID-19 (COV) and long COVID (LC) patients.

COV		% of positive CD169				% of positive HLA-DR				% of positive HLA-DR^+^CD169^+^			
		Leukocytes	Lymphocytes	Monocytes	Granulocytes	Leukocytes	Lymphocytes	Monocytes	Granulocytes	Leukocytes	Lymphocytes	Monocytes	Granulocytes
aPTT ratio	**Rho**	−0.177	−0.060	−0.402^*^	0.045	0.126	0.265	−0.270	0.177	−0.155	−0.025	−0.365^*^	0.235
	***p***	0.275	0.715	**0.010**	0.781	0.439	0.099	0.093	0.273	0.340	0.877	**0.020**	0.145
Azotemia	**Rho**	−0.294^*^	0.281	−0.189	−0.126	−0.046	0.228	0.016	0.206	−0.184	0.329^*^	−0.165	0.017
	***P***	**0.042**	0.053	0.198	0.394	0.756	0.119	0.914	0.160	0.210	**0.022**	0.261	0.909
AST	**Rho**	−0.238	0.031	−0.176	−0.140	−0.390^**^	−0.171	−0.264	−0.066	−0.230	0.027	−0.220	−0.254
	***P***	0.104	0.836	0.232	0.342	**0.006**	0.246	0.070	0.656	0.116	0.854	0.134	0.081
ALT	**Rho**	−0.173	−0.082	−0.087	−0.227	−0.354^*^	−0.174	−0.104	−0.225	−0.232	−0.167	−0.167	−0.423^**^
	***P***	0.239	0.581	0.558	0.121	**0.014**	0.238	0.481	0.124	0.113	0.257	0.256	**0.003**
CRP	**Rho**	−0.068	0.097	−0.116	0.018	−0.232	−0.067	−0.187	0.305^*^	−0.033	0.158	−0.108	−0.024
	***p***	0.656	0.527	0.447	0.907	0.125	0.661	0.219	**0.042**	0.829	0.299	0.479	0.877
LC														
Platelets	**Rho**	0.650^**^	0.057	0.605^**^	−0.096	0.084	−0.223	0.462^*^	0.295	0.638^**^	−0.080	0.592^**^	0.316
	***p***	**0.001**	0.795	**0.002**	0.663	0.703	0.307	**0.027**	0.172	**0.001**	0.717	**0.003**	0.141
PT %	**Rho**	−0.237	0.415^*^	−0.258	0.223	−0.405	0.196	−0.489^*^	−0.324	−0.324	0.496^*^	−0.239	−0.275
	***p***	0.277	**0.049**	0.235	0.306	0.055	0.370	**0.018**	0.131	0.131	**0.016**	0.273	0.203
PT-INR	**Rho**	0.263	**−0.413**	0.281	−0.246	0.396	−0.208	0.529^**^	**0.363**	0.363	−0.499^*^	0.270	0.291
	***p***	0.225	**0.050**	0.193	0.259	0.061	0.340	**0.009**	**0.088**	0.088	**0.015**	0.213	0.178
PT sec	**Rho**	0.126	−0.424^*^	0.174	−0.168	0.404	−0.107	0.383	0.285	0.224	−0.448^*^	0.180	0.248
	***p***	0.565	**0.044**	0.428	0.443	0.056	0.628	0.071	0.188	0.305	**0.032**	0.411	0.255
ALT	**Rho**	0.158	0.027	0.069	−0.104	−0.439^*^	−0.440^*^	0.123	0.065	0.144	−0.036	−0.015	0.221
	***p***	0.473	0.904	0.754	0.636	**0.036**	**0.036**	0.576	0.768	0.512	0.870	0.945	0.310
D-dimer	**Rho**	0.185	0.085	0.031	0.050	0.156	0.105	0.352	0.427^*^	0.226	0.037	0.042	0.253
	***p***	0.399	0.700	0.890	0.819	0.477	0.634	0.100	**0.042**	0.300	0.868	0.847	0.244

Correlations are shown for leukocytes, lymphocytes, monocytes, and granulocytes separately. Parameters analyzed include coagulation markers (aPTT ratio, PT%, PT-INR, PT sec, D-dimer), kidney and liver function (azotemia, AST, ALT), inflammation (CRP), and platelet count. Positive correlations are highlighted in red and negative correlations in blue. Only statistically significant correlations (*p* < 0.05) are color-coded.

aPTT ratio, activated partial thromboplastin time ratio (coagulation marker); PT%, prothrombin time, expressed as percentage; PT-INR, prothrombin time—international normalized ratio; PT sec, prothrombin time in seconds; Platelets, platelet count (cells/μL); azotemia, blood urea nitrogen level (marker of renal function); AST, aspartate aminotransferase (marker of liver and tissue damage); ALT, alanine aminotransferase (marker of liver damage); CRP, C-reactive protein (inflammatory marker); D-dimer, fibrin degradation product; marker of thrombosis and coagulation activation.

Interestingly, in LC, several parameters associated to coagulation correlated with activation marker, particularly with CD169^+^ leukocytes (Rho = 0.650, *p* = 0.001), CD169^+^ monocytes (Rho = 0.605, *p* = 0.002), and HLA-DR^+^CD169^+^ leukocytes (Rho = 0.638, *p* = 0.001), as well as with HLA-DR^+^CD169^+^ monocytes (Rho = 0.592, *p* = 0.003). Furthermore, HLA-DR^+^ monocytes showed a significant positive correlation with PT-INR (Rho = 0.529, *p* = 0.009) and a trend with PT% (Rho = –0.489, *p* = 0.018), both indicating associations with altered coagulation profiles. Similarly, PT sec was negatively correlated with CD169^+^ lymphocytes (Rho = –0.424, *p* = 0.044) and HLA-DR^+^CD169^+^ leukocytes (Rho = –0.448, *p* = 0.032), suggesting immune-mediated changes in coagulation time. In terms of liver markers, a negative correlation was detected between ALT levels and both HLA-DR^+^ lymphocytes (Rho = –0.440, *p* = 0.036) and HLA-DR^+^ leukocytes (Rho = –0.439, *p* = 0.036), potentially indicating immune involvement in residual hepatic inflammation. Finally, D-dimer levels correlated positively with HLA-DR^+^ granulocytes (Rho = 0.427, *p* = 0.042) in LC patients, pointing to sustained low-grade endothelial or coagulation pathway activation. These findings suggest a persistent link between immune activation and coagulation dynamics in acute and post-acute phases.

### 3.6 Correlations of CD169 and HLA-DR-positive EVs with biochemical parameters in COV and LC

Statistical analysis using Spearman’s correlation revealed several significant associations between biochemical parameters and extracellular vesicle subpopulations expressing HLA-DR and CD169 markers in COV patients and in LC individuals (**Tables 6A, B**). In COV, a significant correlation between the percentage and the number of HLA-DR^+^CD169^+^ EVs and azotemia, potassium, and D-dimer was found.

**Table 6:** Spearman correlations between the percentage of extracellular vesicles (EVs) positive for HLA-DR and/or CD169 (A) or EV concentration (events/μL of plasma) and various biochemical and hematological parameters in COVID-19 (COV) and long COVID (LC) patients.

A) % of positive EVs		Total EVs			100_200 nm			240_500 nm			>500 nm		
COV		HLA-DR^+^CD169^−^	HLA-DR^−^CD169^+^	HLA-DR^+^CD169^+^	HLA-DR^+^CD169^−^	HLA-DR^−^CD169^+^	HLA-DR^+^CD169^+^	HLA-DR^+^CD169^−^	HLA-DR^−^CD169^+^	HLADR^+^CD169^+^	HLA-DR^+^CD169^−^	HLA-DR^−^CD169^+^	HLA-DR^+^CD169^+^
**Azotemia**	**Rho**	−0.281	0.099	−0.062	−0.085	−0.070	0.079	−0.206	0.146	−0.040	−0.294^*^	0.079	−0.080
	***p***	0.053	0.503	0.677	0.564	0.635	0.593	0.159	0.321	0.789	**0.043**	0.595	0.590
**Potassium**	**Rho**	−0.285^*^	0.017	−0.065	−0.311^*^	0.005	−0.069	−0.187	0.031	−0.024	−0.315^*^	0.050	−0.094
	***p***	**0.050**	0.909	0.662	**0.032**	0.974	0.640	0.204	0.837	0.870	**0.029**	0.735	0.524
**LC**													
**PT%**	**Rho**	0.026	0.047	0.192	0.457^*^	0.087	0.057	0.090	0.022	0.222	0.164	0.036	0.197
	***p***	0.908	0.831	0.381	**0.028**	0.694	0.796	0.684	0.920	0.308	0.455	0.870	0.367
**PTSec**	**Rho**	−0.120	−0.119	−0.323	−0.446^*^	−0.028	−0.154	−0.205	−0.117	−0.363	−0.242	−0.146	−0.314
	***p***	0.587	0.589	0.133	**0.033**	0.898	0.484	0.348	0.594	0.089	0.266	0.507	0.145
**Azotemia**	**Rho**	0.439	0.047	0.154	0.322	0.157	0.470	0.542^*^	−0.045	0.023	0.445	0.083	0.093
	***p***	0.078	0.858	0.554	0.207	0.548	0.057	**0.025**	0.864	0.929	0.073	0.752	0.724
**LDH**	**Rho**	−0.073	−0.764^**^	−0.491	−0.436	0.418	−0.064	−0.223	−0.755^**^	−0.624^*^	−0.174	−0.636^*^	−0.491
	***p***	0.832	0.006	0.125	0.180	0.201	0.853	0.509	**0.007**	0.040	0.610	**0.035**	0.125
**ALT**	**Rho**	0.518^*^	0.020	0.314	0.345	0.115	0.282	0.428^*^	−0.003	0.315	0.486^*^	0.138	0.284
	***p***	**0.011**	0.927	0.145	0.107	0.601	0.192	**0.042**	0.988	0.143	**0.019**	0.529	0.188
**CRP**	**Rho**	0.346	−0.616^**^	−0.240	−0.069	0.274	0.304	0.411	−0.529^**^	−0.177	0.308	−0.415^*^	−0.295
	***p***	0.106	0.002	0.270	0.756	0.206	0.158	0.052	**0.009**	0.420	0.152	**0.049**	0.172
**SII**	**Rho**	0.487^*^	−0.252	0.001	0.098	0.067	0.212	0.517^*^	−0.264	0.050	0.321	−0.115	−0.042
	***p***	**0.018**	0.246	0.996	0.657	0.762	0.330	**0.011**	0.223	0.819	0.135	0.601	0.851
**NLR**	**Rho**	0.438^*^	−0.309	−0.128	0.059	0.243	0.296	0.468^*^	−0.305	−0.089	0.327	−0.179	−0.175
	***p***	**0.037**	0.151	0.562	0.788	0.264	0.170	**0.024**	0.156	0.686	0.128	0.413	0.425
**PLR**	**Rho**	0.480^*^	−0.182	0.197	0.117	0.127	0.518^*^	0.530^**^	−0.166	0.215	0.338	−0.042	0.144
	***p***	0.020	0.406	0.368	0.596	0.564	**0.011**	**0.009**	0.449	0.325	0.115	0.851	0.511

B) EVs/μL of plasma)		Total EVs			100_200 nm			240_500 nm			>500 nm		
COV		HLA-DR^+^CD169^−^	HLA-DR^−^CD169^+^	HLA-DR^+^CD169^+^	HLA-DR^+^CD169^−^	HLA-DR^−^CD169^+^	HLA-DR^+^CD169^+^	HLA-DR^+^CD169^−^	HLA-DR^−^CD169^+^	HLADR^+^CD169^+^	HLA-DR^+^CD169^−^	HLA-DR^−^CD169^+^	HLA-DR^+^CD169^+^
**D-dimer**	**Rho**	0.039	0.033	−0.009	−0.438^**^	−0.455^**^	−0.375^*^	0.026	0.024	−0.036	0.054	0.063	0.009
	***p***	0.812	0.841	0.955	**0.005**	**0.003**	**0.017**	0.873	0.885	0.824	0.740	0.700	0.956
LC													
**PT%**	**Rho**	−0.427^*^	−0.384	−0.396	0.324	0.179	0.269	−0.402	−0.402	−0.398	−0.454^*^	−0.434^*^	−0.462^*^
	***p***	**0.042**	0.071	0.062	0.132	0.413	0.215	0.057	0.057	0.060	**0.029**	**0.039**	**0.026**
**PT-INR**	**Rho**	0.467^*^	0.432^*^	0.439^*^	−0.330	−0.181	−0.270	0.445^*^	0.448^*^	0.443^*^	0.488^*^	0.472^*^	0.495^*^
	***p***	**0.025**	**0.039**	**0.036**	0.124	0.407	0.213	**0.033**	**0.032**	**0.034**	**0.018**	**0.023**	**0.016**
**CRP**	**Rho**	0.439^*^	0.422^*^	0.410	−0.008	−0.039	−0.034	0.410	0.403	0.375	0.410	0.403	0.397
	***p***	**0.036**	**0.045**	0.052	0.969	0.860	0.876	0.052	0.056	0.078	0.052	0.057	0.061
**NLR**	**Rho**	0.417^*^	0.415^*^	0.415^*^	−0.106	−0.073	−0.018	0.390	0.395	0.381	0.368	0.360	0.349
	***p***	**0.048**	**0.049**	**0.049**	0.631	0.740	0.936	0.066	0.062	0.073	0.084	0.092	0.103

EVs were grouped by size (100–200, 240–500, and >500 nm) and marker expression (HLA-DR^+^CD169^-^, HLA-DR^-^CD169^+^, HLA-DR^+^CD169^+^). Positive correlations are shown in red and negative in blue and wrote in bold. Only statistically significant correlations (*p* < 0.05) are color-coded. Rho: Spearman correlation coefficient; *p*: *p*-value.

EVs, extracellular vesicles; COV, COVID-19 patients; LC, long COVID patients; HLA-DR, human leukocyte antigen-DR isotype (marker of antigen-presenting cells); CD169, cluster of differentiation 169 (Siglec-1, marker of activated myeloid cells); PT%r, prothrombin time (percentage); PTsec, prothrombin time (seconds); PT-INR, prothrombin time-international normalized ratio; LDH, lactate dehydrogenase (marker of tissue damage); ALT, alanine; Aminotransferase (marker of liver function); CRP, C-reactive protein (inflammatory marker); SII, systemic immune-inflammation index; NLR, neutrophil-to-lymphocyte ratio; PLR, platelet-to-lymphocyte ratio; D-dimer, fibrin degradation product (marker of coagulation and thrombosis); Azotemia, elevated blood urea nitrogen (renal function marker); Potassium, serum potassium level (electrolyte balance).

In LC individuals, Spearman correlation analysis revealed more significant associations between different HLA-DR^+^ and CD169^+^ EV subtypes and biochemical parameters. Particularly, a positive correlation with coagulation factors such as PT% and PT-INR, with inflammatory parameters (CRP, SII, NLR, and PLR) and hepatic function parameters (LDH, ALT) (**Tables 6A, B**).

### 3.7 Evaluation of plasmatic cytokines and soluble immune mediators, endothelial activation markers, and neuronal damage indicators in COV and LC

The quantification of soluble immune mediators, endothelial activation markers, and neuronal damage indicators in the plasma of COV patients, LC individuals, and HD controls was performed by ELLA. The median values of different cytokines, such as IL-2, IL-4, CXCL10, IL-6, IL-8, TNF-α, NFL, GMCSF, CCL2, endothelin-1, ST2, TNFR1, IL-10, IL-17A, IL-18, ICAM, and VCAM (ng/mL), for COV, LC, and HD are reported in **Tables 7A, B**.

**Table 7A:** Cytokine levels and other inflammatory or endothelial markers in healthy donors (HD), COVID-19 (COV) patients, and long COVID (LC) individuals. Concentrations of circulating cytokines (IL-1RA, IL-2, IL-4, CXCL10, IL-6, IL-8, TNF-α, IL-10, IL-17A, and IL-18) measured in plasma samples from healthy donors (HD), patients with acute COVID-19 (COV), and individuals with long COVID (LC). Values are reported as minimum, median, and maximum. Significant differences are indicated with asterisks (***p < 0.001, **p < 0.01, *p < 0.05) for COV vs. HD, and plus signs (+++*p* < 0.001, ++*p* < 0.01, +*p* < 0.05) for LC vs. HD.

Groups		IL-1RA	IL-2	IL-4	CXCL10	IL-6	IL-8	TNF-α	IL-10	IL-17A	IL-18
HD	Min	0.56	0.006	0.05	73.4	0.39	2.92	4.75	0.817	0.0	154
	**Median**	**0.94**	**0.087**	**0.051**	**104**	**1.82**	**7.73**	**8.15**	**1.97**	**0.149**	**294**
	Max	686	0.12	0.051	106	4.81	108	11.6	3.48	1.2	334
COV	Min	384	0.05	0	35.7	1.26	0	8.24	2.12	0.0	183
	**Median**	1,135***	0.32**	**0.03**	906**	**14.2**	**8.21**	**17.0**	12.9**	**0.71**	**381**
	Max	6,134	1.22	0.14	2,567	214	180	82.3	43.3	4.19	784
LC	Min	229	0	0	10.9	0.78	0	7.62	1.12	0	116
	**Median**	447***+++	0.06**+++	**0.060**	116*+++	2.44*+++	**9.35**	14.7***	**2.21+++**	**0.420**	**202+++**
	Max	952	0.24	0.34	653	10.8	241	93.1	6.93	4.01	485

**Table 7B:** Levels of inflammatory and endothelial markers [neurofilament light chains (NFLs), granulocyte–macrophage colony-stimulating factor (GM-CSF), CCL2, endothelin-1, ST2, TNFR1, ICAM, and VCAM] in plasma of the HD, COV, and LC groups. Results are expressed as minimum, median, and maximum values. Asterisks and plus signs denote statistically significant differences as described above.

Groups		NFLs	GMCSF	CCL2	Endothelin-1	ST2	TNFR1	ICAM	VCAM		
HD	Min	0.45	0.0	148	1.08	16,209	910	213,077	462,976		
	**Median**	**1.09**	**0.812**	**174**	**1.22**	**20**,**084**	**942**	**229**,**650**	**489**,**833**		
	Max	25.6	1.12	647	5.07	23,272	1,117	238,020	504,408		
COV	Min	0.0	0.06	113	1.15	9,193	877	222,419	563,897		
	**Median**	**28.1**	**1.66**	**272**	**2.86**	**115**,268**	**2**,790**	**384**,168**	**1**,**166**,051**		
	Max	213	1,463	1,293	6.95	332,013	13,455	930,015	2,723,200		
LC	Min	0	0	258	1.3	8,973	849	185,596	533,435		
	**Median**	**10.7+**	**0.520**	**364+**	**2.66**	**23**,**117+++**	**1**,544*	**451**,218**	**1**,**027**,666**		
	Max	684	298	943	3.78	125,794	9,341	1,456,533	3,450,487		

Analyses showed significantly high serum concentrations (ng/mL) of IL-6 (*p* = 0.000; *p* = 0.033), TNF-α (*p* = 0.000; *p* = 0.000), TNFR (*p* = 0.019; *p* = 0.028), ICAM (*p* = 0.025; *p* = 0.016), and VCAM (*p* = 0.006; *p* = 0.005) in COV and LC compared with HD. Only in the COV group, a higher concentration of IL-2 (*p* = 0.011) and ST2 (*p* = 0.031) was observed with respect to HD.

Furthermore, in COV, a statistically significant difference of IL-2 (*p* = 0.000), CXCL10 (*p* = 0.000), IL-6 (*p* = 0.000), NFL (*p* = 0.047), CCL2 (*p* = 0.038), ST2 (*p* = 0.000), IL-10 (*p* = 0.000), and IL-18 (*p* = 0.000) emerged with respect to LC. No statistically significant differences emerged in the concentrations of IL-4, IL-8, GMCSF, endothelin-1, and IL-17A between the different groups.

### 3.8 Circulating cytokines correlate with CD169 and HLA-DR-positive leukocytes in COV and LC

The association between CD169 markers in leukocytes with cytokines and inflammatory mediators (pg/mL) was tested through the Spearman correlation analysis (**Table 8**). In the COV group, a negative correlation between leukocytes, monocytes, and granulocytes expressing HLA-DR and CD169 and most cytokines such as IL-1RA, IL-8, and TNFR1 was found. Interestingly, in LC individuals, a strong and positive correlation emerged between the different HLA-DR^+^CD169^+^ leukocyte subgroups with different cytokines, particularly monocytes with CXCL10, IL-10, and IL-8.

**Table 8:** Spearman correlations between the percentage of immune cells positive for CD169, HLA-DR, or both (HLA-DR^+^CD169^+^) and cytokines in COVID-19 (COV) and long COVID (LC) patients.

COV		% of positive CD169				% of positive HLA-DR				% of positive HLA-DR+CD169+			
		Leukocytes	Lymphocytes	Monocytes	Granulocytes	Leukocytes	Lymphocytes	Monocytes	Granulocytes	Leukocytes	Lymphocytes	Monocytes	Granulocytes
IL-1RA	Rho	−0.154	0.213	−0.358	−0.095	−0.642**	0.184	−0.514^*^	0.219	−0.268	0.196	−0.349	−0.314
	*p*	0.528	0.382	0.132	0.700	**0.003**	0.450	**0.024**	0.367	0.267	0.420	0.143	0.191
IL-2	Rho	−0.150	0.106	−0.230	−0.015	−0.734^**^	−0.109	−0.530^*^	0.162	−0.232	0.011	−0.200	−0.096
	*p*	0.539	0.666	0.343	0.952	**0.000**	0.657	**0.020**	0.508	0.339	0.963	0.413	0.695
IL-8	Rho	−0.127	0.105	−0.320^*^	0.033	−0.107	0.129	−0.082	0.045	−0.154	0.103	−0.276	−0.090
	*p*	0.394	0.482	**0.028**	0.828	0.475	0.389	0.584	0.766	0.301	0.492	0.060	0.546
NFLs	Rho	−0.229	0.016	−0.314^*^	−0.129	−0.033	0.037	−0.044	0.136	−0.158	0.064	−0.262	0.003
	*p*	0.122	0.915	**0.032**	0.387	0.824	0.804	0.770	0.360	0.290	0.667	0.075	0.983
CCL2	Rho	−0.195	0.155	−0.191	−0.130	−0.654^**^	0.423	−0.193	0.170	−0.181	0.223	−0.181	−0.325
	*p*	0.424	0.525	0.433	0.596	**0.002**	0.071	0.429	0.486	0.459	0.359	0.459	0.175
ST2	Rho	−0.289	0.161	−0.426	−0.198	−0.253	0.067	−0.265	0.314	−0.372	0.154	−0.467^*^	−0.162
	*p*	0.229	0.511	0.069	0.416	0.297	0.786	0.273	0.190	0.117	0.528	**0.044**	0.506
TNFR1	Rho	−0.442	−0.006	−0.619^**^	−0.228	−0.530^*^	−0.046	−0.435	0.402	−0.449	0.037	−0.582^**^	−0.134
	*p*	0.058	0.980	**0.005**	0.348	**0.020**	0.853	0.063	0.088	0.054	0.881	**0.009**	0.583
IL-10	Rho	−0.123	0.261	−0.326	−0.216	−0.668^**^	0.112	−0.532^*^	0.040	−0.202	0.218	−0.312	−0.254
	*p*	0.616	0.281	0.173	0.375	**0.002**	0.647	**0.019**	0.870	0.408	0.371	0.193	0.294
IL-18	Rho	0.181	−0.032	0.025	−0.093	−0.286	0.047	0.011	−0.188	0.039	0.021	−0.018	−0.546^*^
	*p*	0.459	0.898	0.920	0.705	0.235	0.847	0.966	0.442	0.875	0.932	0.943	**0.016**
VCAM	Rho	−0.207	0.061	−0.400	0.189	−0.514^*^	−0.149	−0.353	0.342	−0.363	0.019	−0.405	−0.047
	*p*	0.395	0.803	0.090	0.437	**0.024**	0.542	0.139	0.152	0.126	0.937	0.085	0.850
LC													
IL-1β	Rho	0.306	0.212	0.239	0.129	0.581^**^	0.131	0.353	0.565^**^	0.246	0.011	0.165	0.377
	*p*	0.145	0.319	0.260	0.547	**0.003**	0.543	0.091	**0.004**	0.246	0.958	0.441	0.069
IL-1RA	Rho	0.127	0.265	0.007	0.287	0.060	0.252	0.340	0.545^**^	0.165	0.284	0.079	0.412^*^
	*p*	0.553	0.211	0.974	0.174	0.779	0.235	0.104	**0.006**	0.441	0.179	0.713	**0.046**
CXCL10	Rho	0.517^**^	0.147	0.459^*^	0.016	0.450^*^	−0.011	0.498^*^	0.317	0.508^*^	0.028	0.455^*^	0.289
	*p*	**0.010**	0.494	**0.024**	0.942	**0.027**	0.958	**0.013**	0.131	**0.011**	0.897	**0.025**	0.170
IL-8	Rho	0.614^**^	−0.178	0.525^**^	−0.275	0.239	−0.348	0.775^**^	0.506^*^	0.657^**^	−0.347	0.565^**^	0.304
	*p*	**0.001**	0.405	**0.008**	0.194	0.260	0.096	**0.000**	**0.012**	**0.000**	0.097	**0.004**	0.149
TNF-α	**Rho**	−0.169	0.066	−0.126	0.318	0.479^*^	0.540^**^	0.136	0.325	−0.120	0.272	−0.077	0.172
	*p*	0.431	0.759	0.557	0.130	**0.018**	**0.006**	0.527	0.121	0.578	0.198	0.722	0.421
GMCS	Rho	−0.271	0.514^*^	−0.189	0.667^**^	−0.098	0.441^*^	−0.481^*^	−0.088	−0.310	0.616^**^	−0.243	0.290
	*p*	0.201	**0.010**	0.377	**0.000**	0.649	**0.031**	**0.017**	0.682	0.141	**0.001**	0.253	0.169
TNFR1	*Rho*	0.381	0.183	0.205	0.134	0.188	0.149	0.583^**^	0.493^*^	0.344	0.117	0.198	0.425^*^
	*p*	0.067	0.392	0.336	0.533	0.379	0.488	**0.003**	**0.014**	0.099	0.585	0.353	**0.038**
IL-10	Rho	0.553^**^	0.020	0.514^*^	0.045	0.215	0.064	0.539^**^	0.413^*^	0.584^**^	0.004	0.568^**^	0.362
	*p*	**0.005**	0.927	**0.010**	0.835	0.313	0.765	**0.007**	**0.045**	**0.003**	0.984	**0.004**	0.082
IL-17A	Rho	0.234	0.002	0.277	0.144	0.116	0.039	0.304	0.239	0.398	0.145	0.426^*^	0.394
	*p*	0.270	0.993	0.190	0.501	0.591	0.856	0.149	0.261	0.054	0.500	**0.038**	0.057

Correlations are shown for leukocytes, lymphocytes, monocytes, and granulocytes separately and the levels of selected cytokines and soluble markers in COV (IL-1RA, IL-2, IL-8, NFLs, CCL2, ST2, TNFR1, IL-10, IL-18, VCAM) and in LC (IL1-β, IL-1RA, CXCL10, IL-8, TNF-α, GMCS, TNFR1, IL-10, IL-17A). Positive correlations are highlighted in red and negative correlations in blue. Only statistically significant correlations (*p* < 0.05) are color-coded.

### 3.9 Circulating cytokines correlate with CD169 and HLA-DR-positive EVs in COV and LC

In COV, as reported in **Tables 9A,B**, significant correlations were observed between specific EV subpopulations and circulating cytokines and inflammatory mediators. IL-8 (pg/mL) levels showed a positive correlation with both the percentage of HLA-DR^+^CD169^+^ EVs (*ρ* = 0.316, *p* = 0.031) and the concentration of HLA-DR^+^ EVs larger than 500 nm (Rho = 0.353, *p* = 0.015), suggesting an association between IL-8-mediated inflammation and increased presence of immune-activated EVs. Also, a negative correlation with the percentages of the HLA-DR^+^CD169^+^ EV subgroup and TNF-α, endothelin-1, IL-10, and ST2 emerged. Moreover, considering the number of positive EVs, the analysis showed a negative correlation only with CCL2.

**Table 9:** Spearman correlations between extracellular vesicles (EVs) and circulating cytokine levels in COVID-19 (COV) and long COVID (LC) patients.

A) % of positive EVs		Total EVs			100_200 nm			240_500 nm			>500 nm		
COV		HLA-DR^+^CD169^−^	LA-DR^−^CD169^+^	HLA-DR^+^CD169^+^	HLA-DR^+^CD169^−^	HLA-DR^−^CD169^+^	HLA-DR^+^CD169^+^	HLA-DR^+^CD169^−^	HLA-DR^−^CD169^+^	HLA-DR^+^CD169+	HLA-DR^+^CD169^−^	HLA-DR^−^CD169^+^	HLA-DR^+^CD169^+^
**IL-8**	**Rho**	−0.103	0.048	0.316^*^	−0.091	−0.184	0.105	−0.048	0.032	0.281	−0.128	0.027	0.353^*^
	***p***	0.490	0.751	**0.031**	0.542	0.215	0.484	0.747	0.832	0.056	0.391	0.854	**0.015**
**TNF-α**	**Rho**	−0.230	−0.057	0.066	−0.314^*^	−0.157	0.172	−0.153	−0.007	0.052	−0.225	−0.074	0.109
	***p***	0.119	0.701	0.660	**0.031**	0.290	0.248	0.305	0.962	0.728	0.128	0.622	0.465
**Endothelin-1**	**Rho**	0.099	0.346	0.427	−0.465^*^	−0.111	0.409	0.275	0.468^*^	0.446	−0.037	0.240	0.300
	***p***	0.686	0.147	0.069	**0.045**	0.652	0.082	0.255	**0.043**	0.056	0.881	0.323	0.212
**ST2**	**Rho**	−0.398	0.112	−0.011	−0.278	−0.123	0.032	−0.153	0.230	0.006	−0.532^*^	0.095	−0.023
	***p***	0.092	0.647	0.963	0.249	0.616	0.898	0.532	0.344	0.980	**0.019**	0.700	0.926
**IL-10**	**Rho**	−0.456^*^	−0.081	0.149	−0.254	0.270	−0.164	−0.280	0.029	0.147	−0.500^*^	−0.118	0.126
	***p***	**0.050**	0.743	0.542	0.293	0.263	0.502	0.246	0.906	0.549	**0.029**	0.632	0.606
LC													
**IL-2**	**Rho**	−0.037	−0.257	−0.342	0.428^*^	0.385	−0.173	−0.101	−0.432^*^	−0.254	0.030	−0.271	−0.353
	***p***	0.859	0.215	0.094	**0.033**	0.057	0.409	0.630	**0.031**	0.220	0.886	0.190	0.083
**IL-4**	**Rho**	0.206	−0.026	0.061	0.112	−0.106	0.398^*^	0.315	−0.218	0.110	0.050	0.130	−0.002
	***p***	0.323	0.900	0.771	0.596	0.613	**0.049**	0.125	0.295	0.601	0.813	0.535	0.994
**CXCL10**	**Rho**	−0.154	0.296	0.427^*^	−0.127	−0.443^*^	0.158	−0.089	0.313	0.463^*^	−0.223	0.463^*^	0.389
	***p***	0.464	0.151	**0.033**	0.544	**0.027**	0.450	0.673	0.127	**0.020**	0.283	**0.020**	0.055
**CCL2**	**Rho**	0.373	−0.008	0.179	0.362	0.055	−0.033	0.386	−0.030	0.209	0.405^*^	−0.001	0.171
	***p***	0.066	0.971	0.391	0.076	0.794	0.875	0.057	0.885	0.315	**0.045**	0.997	0.414
**Endothelin-1**	**Rho**	0.088	−0.182	−0.148	0.162	0.472*	−0.060	0.080	−0.217	−0.090	0.064	−0.155	−0.202
	***p***	0.675	0.385	0.480	0.440	**0.017**	0.774	0.705	0.297	0.669	0.760	0.459	0.333
**ST2**	**Rho**	0.493^*^	−0.179	0.200	0.094	−0.260	0.171	0.455^*^	−0.122	0.158	0.348	−0.020	0.152
	***p***	**0.012**	0.391	0.338	0.655	0.209	0.414	**0.022**	0.563	0.451	0.088	0.924	0.467
**TNFR1**	**Rho**	0.402^*^	−0.116	0.119	0.280	−0.043	0.160	0.347	−0.244	0.125	0.379	0.017	0.088
	***p***	**0.047**	0.580	0.571	0.175	0.837	0.445	0.089	0.241	0.552	0.061	0.936	0.674

B) EVs/μL of plasma		Total EVs			100_200 nm			240_500 nm			>500 nm		
COV		HLA-DR^+^CD169^−^	HLA-DR^−^CD169^+^	HLA-DR^+^CD169^+^	HLA-DR^+^CD169^−^	HLA-DR^−^CD169^+^	HLA-DR^+^CD169^+^	HLA-DR^+^CD169^−^	HLA-DR^−^CD169^+^	HLA-DR^+^CD169^+^	HLA-DR^+^CD169-	HLA-DR^−^CD169^+^	HLA-DR^+^CD169^+^
**IL-8**	**Rho**	−0.246	−0.256	−0.215	−0.002	−0.052	0.009	−0.237	−0.222	−0.189	−0.260	−0.237	−0.201
	***p***	0.095	0.083	0.147	0.990	0.727	0.953	0.108	0.134	0.204	0.078	0.109	0.176
**TNF-α**	**Rho**	−0.106	−0.077	−0.061	−0.138	−0.148	−0.060	−0.077	−0.042	−0.045	−0.094	−0.068	−0.045
	***p***	0.479	0.608	0.685	0.355	0.319	0.686	0.605	0.777	0.766	0.529	0.651	0.765
**CCL2**	**Rho**	−0.458^*^	−0.463^*^	−0.428	0.167	0.153	0.068	−0.486^*^	−0.460^*^	−0.474^*^	−0.433	−0.454	−0.404
	***p***	**0.049**	**0.046**	0.067	0.495	0.533	0.783	**0.035**	**0.048**	**0.040**	0.064	0.051	0.087
**Endothelin-1**	**Rho**	−0.319	−0.323	−0.323	−0.146	0.005	0.210	−0.340	−0.314	−0.330	−0.288	−0.316	−0.365
	***p***	0.183	0.178	0.178	0.552	0.983	0.389	0.154	0.190	0.168	0.232	0.188	0.124
**ST2**	**Rho**	−0.351	−0.288	−0.351	−0.144	−0.047	−0.122	−0.326	−0.216	−0.342	−0.342	−0.347	−0.388
	***p***	0.141	0.232	0.141	0.557	0.847	0.619	0.173	0.375	0.152	0.152	0.145	0.101
**IL-10**	**Rho**	−0.340	−0.291	−0.328	0.128	0.314	0.140	−0.339	−0.225	−0.318	−0.321	−0.321	−0.314
	***p***	0.154	0.226	0.170	0.601	0.190	0.569	0.156	0.355	0.185	0.180	0.180	0.190
LC													
**CXCL10**	**Rho**	−0.115	−0.121	−0.113	−0.324	−0.430^*^	−0.250	−0.127	−0.123	−0.117	−0.071	−0.084	−0.067
	***p***	0.585	0.564	0.589	0.114	**0.032**	0.227	0.544	0.558	0.576	0.737	0.690	0.751
**Endothelin-1**	**Rho**	0.405^*^	0.436^*^	0.415^*^	0.046	0.066	−0.021	0.419^*^	0.447^*^	0.428^*^	0.413^*^	0.403^*^	0.418^*^
	***p***	0.045	**0.029**	**0.039**	0.828	0.755	0.920	**0.037**	**0.025**	**0.033**	**0.040**	**0.046**	**0.038**

**Table 9A** reports correlations between the percentage of EVs expressing HLA-DR and/or CD169 and the levels of selected cytokines and soluble markers (IL-8, TNF-α, CCL2, endothelin-1, ST2, IL-10 in COV; IL-2, IL-4, CXCL10, CCL2, endothelin-1, ST2, TNFR1 in LC). **Table 9B** shows correlations between absolute EV counts (EVs/µL of plasma) and the same cytokines. EVs were analyzed based on size classes (100–200, 240–500, >500 nm) and surface marker expression (HLA-DR^+^CD169^-^, HLA-DR^-^CD169^+^, HLA-DR^+^CD169^+^). Positive correlations are highlighted in red and negative correlations in blue. Only statistically significant correlations (*p* < 0.05) are color-coded.

In LC, the analysis revealed several statistically significant correlations between circulating cytokines and distinct subsets of EVs stratified by surface markers (HLA-DR and CD169) and size (100–200, 240–500, and >500 nm). Specifically, a positive correlation between the percentage of EVs in different subgroups and IL-4, CCL2, endothelin-1, ST2, and TNFR1 was found. Particularly, the percentage of HLA-DR^+^CD169^+^ EVs showed a positive correlation with CXCL10, a chemokine associated with interferon signaling and Th1-type immune responses, and endothelin-1, a marker of endothelial dysfunction.

### 3.10 Distribution of EV-associated immune cell- and platelet-derived markers in HD, COV, and LC

The analysis of EV-associated markers revealed marked quantitative differences between HD, COV, and LC, affecting both the relative frequency (% of positive EVs) and the absolute number of EV-positive events per microliter (**Table 10A**). Monocyte-derived EVs in HD and EVs expressing classical (CD14) and non-classical (CD16) monocyte markers showed relatively high baseline frequencies, with median values of 1.80% and 3.89%, respectively. In contrast, COV patients exhibited a marked reduction in CD14^+^ and CD16^+^ EV frequencies, with median values of 2.35% and 1.20%. This reduction persisted in LC, where CD14^+^ EVs were further decreased (median 0.49%), while CD16 EVs partially recovered (median 4.04%). Notably, CD169^+^ EVs, reflecting interferon-activated monocytes, were low in HD (median 1.31%) but dramatically increased in COV (median 12.28%, *p* < 0.01) and remained elevated in LC (median 11.53%). This pattern was even more evident when considering absolute counts, with CD169^+^ EV events increasing from a median of 31.54 events/µL in HD to 243.96 events/µL in COV and remaining high in LC (128.47 events/µL). In lymphocyte-derived EVs, those expressing T-cell markers (CD3, CD4, CD8) were present at low frequencies in HD, consistent with immune homeostasis. In COV, these EV subsets were significantly reduced, particularly CD3^+^ and CD4^+^ EVs (median 0.03% and 0.41%, *p* < 0.01), reflecting acute lymphocyte dysfunction or redistribution. In LC, however, a rebound and selective enrichment of CD4^+^ EVs was observed (median 15.25%), accompanied by increased absolute counts (median 86.52 events/µL), suggesting persistent or compensatory T-cell activation after acute infection. CD8^+^ EVs also showed higher frequencies and counts in LC compared with both HD and COV. In natural killer (NK) cell-derived EVs, NK-associated EVs showed moderate levels in HD (median 4.54%), while COV patients exhibited a marked increase (median 12.38%), which persisted in LC (median 13.75%). The absolute NK EV counts followed a similar trend, increasing from 84.47 events/µL in HD to 233.26 events/µL in COV and 263.57 events/µL in LC, indicating sustained innate immune activation beyond the acute phase. Platelet-derived EVs expressing CD61 were abundant in HD (median 17.00%) and decreased in COV (median 10.14%), followed by partial recovery in LC (median 16.34%). In contrast, EVs expressing the activation markers CD41 and CD62P were markedly increased in COV, with CD62P^+^ EVs rising from 2.43% in HD to 7.47% in COV and remaining elevated in LC (6.87%). Absolute counts confirmed these findings, with CD62P^+^ EV events nearly doubling in COV compared with HD and remaining higher than baseline in LC, consistent with platelet activation and prothrombotic signaling.

**Table 10A:** Percentage of positivity and events of cell-derived markers in EVs from the plasma of the HD, COV, and LC groups.

Markers			Monocyte-derived			Lymphocyte-derived			Natural killer-derived	Platelet-derived		
% of positive EVs			CD14	CD16	CD169	CD3	CD4	CD8	NK	CD61	CD41	CD62P
HD	**Median**		**1.80%**	**3**.**89%**	**1**.**31%**	**0**.**71%**	**1**.**38%**	**3**.**13%**	**4**.**54%**	**17**.**00%**	**0**.**37%**	**2**.**43%**
	IQR	25	0.51%	2.04%	0.19%	0.52%	0.93%	1.00%	1.34%	15.87%	0.20%	2.27%
		50	1.80%	3.89%	1.31%	0.71%	1.38%	3.13%	4.54%	17.00%	0.37%	2.43%
		75	14.43%	8.18%	7.46%	3.77%	25.74%	15.22%	41.48%	30.27%	10.92%	6.79%
COV	**Median**		**2**.**35%**	**1**.**20%**	**12**.28%**	**0**.03%**	**0**.41%**	**1**.**15%**	**12**.**38%**	**10**.**14%**	**0**.**28%**	**7**.**47%**
	IQR	25	1.92%	1.09%	11.43%	0.02%	0.26%	0.65%	12.01%	6.07%	0.25%	6.11%
		50	2.35%	1.20%	12.28%	0.03%	0.41%	1.15%	12.38%	10.14%	0.28%	7.47%
		75	16.44%	8.45%	37.49%	1.69%	11.49%	17.70%	36.32%	16.92%	3.18%	10.73%
LC	**Median**		**0**.**49%**	**4**.**04%**	**11**.53%**	**0**.**70%**	**15**.**25%**	**4**.**84%**	**13**.**75%**	**16**.**34%**	**0**.**40%**	**6**.**87%**
	IQR	25	0.27%	0.03%	0.42%	0.49%	1.11%	1.17%	2.75%	14.63%	0.19%	1.78%
		50	0.49%	4.04%	11.53%	0.70%	15.25%	4.84%	13.75%	16.34%	0.40%	6.87%
		75	14.31%	5.49%	25.10%	12.07%	19.03%	7.13%	34.42%	20.95%	6.96%	7.83%

Events/μL of positive cells			CD14	CD16	CD169	CD3	CD4	CD8	NK	CD61	CD41	CD62P
HD	**Median**		**51**.**17**	**43**.**34**	**31**.**54**	**12**.**50**	**22**.**47**	**25**.**18**	**84**.**47**	**233**.**32**	**3**.**20**	**39**.**21**
	IQR	25	9.66	36.86	4.14	8.87	15.74	16.83	6.25	146.31	1.27	7.29
		50	51.17	43.34	31.54	12.50	22.47	25.18	84.47	233.32	3.20	39.21
		75	207.19	96.16	79.02	52.65	208.11	152.57	504.33	293.97	49.18	53.83
COV	**Median**		**46**.**70**	**23**.**26**	**243**.**96**	**0**.**38**	**7**.**03**	**19**.**69**	**233**.**26**	**125**.**54**	**5**.**17**	**76**.**43**
	IQR	25	37.49	21.05	168.60	0.18	3.34	9.16	138.67	44.34	4.27	40.26
		50	46.70	23.26	243.96	0.38	7.03	19.69	233.26	125.54	5.17	76.43
		75	238.32	101.17	608.15	22.82	157.78	244.67	464.74	232.69	16.72	145.16
LC	**Median**		**10**.**06**	**37**.**35**	**128**.**47**	**10**.**86**	**86**.**52**	**33**.**80**	**263**.**57**	**157**.**10**	**5**.**99**	**23**.**84**
	IQR	25	0.72	0.07	2.17	4.27	13.45	10.68	82.56	63.37	1.72	9.38
		50	10.06	37.35	128.47	10.86	86.52	33.80	263.57	157.10	5.99	23.84
		75	67.78	49.54	193.48	68.77	155.50	49.30	353.47	289.15	30.92	60.77

Results are expressed as minimum, median, and maximum values. Asterisks denote statistically significant differences as described above.

**Table 10B:** Percentage of positivity and events of SARS-CoV-2 proteins and cytokines in EVs from the plasma of the HD, COV, and LC groups.

				SARS-CoV-2		Chemokine and cytokines		
% of positive EVs			SPIKE RBD	SPIKE S1	CXCL10	IL-10	IL-6	TNF-α
HD	**Median**		**1**.**33%**	**0**.**50%**	**4**.**03%**	**17**.**32**	**24**.**69**	**22**.**27**
	IQR	25	0.99%	0.50%	2.81%	16.00	22.83	20.37
		50	1.33%	0.50%	4.03%	17.32	24.69	22.27
		75	2.19%	7.56%	5.45%	17.61	29.33	24.53
COV	**Median**		**4**.9%**	**7**.06%**	**11**.03%**	**18**.**61**	**37**.24**	**23**.**06**
	IQR	25	3.97%	2.85%	10.08%	16.47	36.23	19.17
		50	4.90%	7.06%	11.03%	18.61	37.24	23.06
		75	9.38%	8.60%	16.59%	19.80	39.05	35.54
LC	**Median**		**6**.41%**	**2**.64%*	**4**.**23%**	**19**.**48**	**36**.79*	**28**.**87**
	IQR	25	2.75%	1.20%	1.02%	18.19	33.97	20.55
		50	6.41%	2.64%	4.23%	19.48	36.79	28.87
		75	8.20%	3.84%	5.33%	21.96	40.01	31.33

**Events/μL of positive cells**			**SPIKE RBD**	**SPIKE S1**	**CXCL10**	**IL-10**	**IL-6**	**TNF-**α
HD	**Median**		**360**.**39**	**136**.**86**	**63**.**04**	**136**.**14**	**103**.**82**	**103**.**82**
	IQR	25	117.87	75.66	19.43	69.51	71.65	71.65
		50	360.39	136.86	63.04	136.14	103.82	103.82
		75	362.87	1,186.40	131.69	176.89	133.09	133.09
COV	**Median**		**529**.**25**	**799.02**	**1,622**.**31**	**157**.**00**	**216**.**85**	**216**.85**
	IQR	25	321.70	580.17	10.75	130.05	174.85	174.85
		50	529.25	799.02	2.31	157.00	216.85	216.85
		75	757.99	1,314.00	285.65	368.67	285.41	285.41
LC	**Median**		**263**.**57**	**495**.**27**	**16**.**17**	**183**.**91**	**187**.**43**	**187**.**43**
	IQR	25	218.20	146.98	8.83	126.07	105.67	105.67
		50	263.57	495.27	16.17	183.91	187.43	187.43
		75	371.51	594.11	29.70	436.46	208.15	208.15

Results are expressed as minimum, median, and maximum values. Asterisks denote statistically significant differences as described above.

### 3.11 EV-associated SARS-CoV-2 antigens and inflammatory mediators in HD, COV, and LC

The analysis of EVs carrying SARS-CoV-2 antigens and immune mediators revealed pronounced differences between the HD, COV, and LC groups, affecting both the relative abundance (% of positive EVs) and absolute EV counts (events/µL) (**Table 10B**). In healthy donors, EVs positive for Spike RBD and Spike S1 were detected at low baseline frequencies (median 1.33% and 0.50%, respectively), consistent with the absence of active or residual viral antigen exposure. In contrast, COV patients exhibited a significant enrichment of Spike-positive EVs, with median frequencies increasing to 4.90% for Spike RBD and 7.06% for Spike S1 (*p* < 0.01). These differences were also evident in absolute counts, with Spike S1 EVs rising from a median of 136.86 events/µL in HD to 799.02 events/µL in COV, indicating substantial viral antigen incorporation into circulating EVs during acute infection. In LC subjects, Spike RBD EVs remained significantly elevated compared with HD (median 6.41%, *p* < 0.01), while Spike S1 EVs showed a partial but still significant increase (median 2.64%, *p* < 0.05). Indeed, the absolute EV counts in LC were lower than in COV but remained above HD levels, supporting the presence of persistent viral antigen signatures beyond the acute phase. EVs expressing CXCL10 (IP-10) were present at moderate levels in HD (median 4.03%). In COV patients, CXCL10 EVs were significantly increased (median 11.03%, *p* < 0.01), with a significant increase in absolute counts (median 1,622.31 events/µL), reflecting intense interferon-driven inflammation during acute disease. In LC, CXCL10 EV frequencies and counts were markedly reduced compared with COV and approached HD values, suggesting partial resolution of acute interferon signaling. EV-associated IL-6 showed a significant increase in COV compared with HD (median 37.24 vs. 24.69, *p* < 0.01), consistent with systemic inflammation. Elevated IL-6 EV levels persisted in LC (median 36.79, *p* < 0.05), indicating ongoing low-grade inflammatory activity. Absolute IL-6 EV counts followed a similar pattern, remaining higher in both COV and LC compared with HD. IL-10-positive EVs showed only moderate increases across groups, with slightly higher medians in COV and LC compared with HD, suggesting activation also of anti-inflammatory pathways. EV-associated TNF-α levels were comparable between HD and COV in relative frequency but showed a significant increase in absolute counts in COV (median 216.85 events/µL, *p* < 0.01). In LC, TNF-α EV counts remained elevated compared with HD, supporting the presence of persistent pro-inflammatory signaling.

### 3.12 Distinct immunological and extracellular vesicle signatures associated with neurological involvement in long COVID

A first stratification comparing LC patients without any symptoms (9/25) to those reporting at least one symptom (16/25) revealed statistically significant differences in neutrophil and lymphocyte percentages, as well as in the inflammatory indices SII and NLR, along with altered potassium levels (**Table 11**). When stratifying patients according to the presence of systemic symptoms, significant differences in IL-1β, IL-1RA, TNF, and IL-10 levels, along with platelet counts, emerged. No significant differences were observed in CD169^+^ and HLA-DR^+^ EV populations in these comparisons, implying that EV-mediated immune signaling may not globally distinguish symptomatic from asymptomatic LC profiles, at least when symptoms are considered in broad categories. However, when focusing on neurological symptoms, a distinct pattern emerged (**Table 11**). Patients reporting neurological manifestations (14/25) showed significantly decreased CRP and potassium levels and, notably, a significant reduction in the concentration (events/µL) of CD169^+^/HLA-DR^+^ EVs, particularly within the >500- and 240–500-nm size ranges (**Table 12**). The EV reduction suggests a selective impairment or altered release of antigen-presenting EVs in neurosymptomatic LC patients. Principal component analysis revealed marked differences in the latent immunological structure of patients with and without neurological symptoms (**Table 13**). In the *no-neuro symptoms* group, the rotated solution identified three compact components: i) a dominant monocyte activation factor characterized by EVs positive for HLA-DR^+^ and/or CD169^+^ subsets; ii) a classical pro-inflammatory cytokine cluster (IL-1β, IL-6, IL-8, GM-CSF, TNF-α); and iii) a hematologic/coagulation-derived inflammation axis driven by leukocyte and platelet indices (NLR, PLR, SII, WBC, platelets). In contrast, *neuro symptom* patients displayed a marked, fragmented immune profile, expanding to six components. Notably, a distinct neuroinflammatory factor emerged, defined by IL-17A, VCAM, and neurofilaments (NFLs). Pro-inflammatory cytokines (IL-1β, TNF-α, CXCL10), GM-CSF/CCL2 myeloid recruitment signals, and acute-phase markers (PCR, NLR, PLR, SII) segregated into independent components, while platelet and white blood cell parameters also formed a separate cluster. Overall, patients with neurological symptoms exhibited a loss of coordinated immune organization, with neuroinflammation-specific signals emerging as independent drivers of variance.

**Table 11:** Comparison of hematological, biochemical, inflammatory and cytokine parameters between Long COVID (LC) individuals without symptoms and those with persistent symptoms.

LC individuals		Lymphocyte s (%)	Neutrophil s (%)	Potassium (mEq/L)	IL1RA (pg/mL)	SII	NLR	
**NO_symptoms**	**Min**	60,8	1,47	3,9	456,6	2,02	229	
	**Median**	65,4	25,6	4,4	698,6	2,5	403	
	**Max**	73,7	30,1	5,1	1106	3,55	513	
**With symptoms**	**Min**	42,1	19,3	3,6	203,6	0,9	334	
	**Median**	54,5 ^*^	34,1^*^	4,1^*^	381,9^*^	1,6^*^	**501**	
	**Max**	69,5	45,3	4,8	967,5	3,5	952	

		platelets	IL1β (pg/mL)	IL1RA (pg/mL)	TNFα (pg/mL)	GMCSF (pg/mL)	CCL2 (pg/mL)	IL-10 (pg/mL)
**NO_symptoms**	**Min**	175	0,23	229	7,62	0	312	1,12
	**Median**	226	0,27	382,5	11	0,05	328,5	1,70
	**Max**	275	0,50	447	17,20	145	733	2,92
**With symptoms**	**Min**	193	0,14	334	10,90	0	258	1,12
	**Median**	275^*^	0,50^*^	513^**^	15,80^*^	1,49^*^	462^*^	
	**Max**	394	1,38	952	93,10	298	943	

Data are reported as minimum (Min), median (Median) and maximum (Max) values. Statistical differences between groups were assessed using the non-parametric Kruskal–Wallis test. Asterisks indicate statistically significant differences between LC individuals with symptoms and those without symptoms (*p* < 0.05; p < 0.01).

**Table 12:** Comparison of biochemical, inflammatory, and extracellular vesicle (EV)-related parameters in long COVID (LC) individuals stratified according to the presence or absence of neurological symptoms.

LC individuals		Potassium (mEq/L)		RCP			SII	
NO_NEUROLOGIC symptoms	**Min**	3,90		1			203,63	
	**Median**	4,40		3			646,71	
	**Max**	5,10		14			1106	
NEUROLOGIC symptoms	**Min**	3,60		1			223,84	
	**Median**	4,10^*^		1^*^			475,90^*^	
	**Max**	4,80		41			967,57	
EVs/µl of plasma		**HLADR+** **CD169+** **(tot)**	**HLA-DR+** **(tot)**	**CD169+** **(tot)**	**CD169+** **> 500 nm**	**HLA-DR** **CD169+** **240-500 nm**	**HLA-DR+** **240-500 nm**	**CD169+** **240-500 nm**
LC NO_NEUROLOGIC symptoms	**Min**	583,26	788,29	988,3	115,83	277,72	378,58	470,67
	**Median**	1174,34	1969,56	2486,65	835,59	598,40	1018,55	1401,76
	**Max**	1801,50	3267,93	4185,36	1670,07	899,64	1528,93	2104,74
LC NEUROLOGIC symptoms	**Min**	227,41	315,86	436,66	164,43	110,98	158,25	231,73
	**Median**	467,61^*^	764,36^*^	986,02^*^	380,74^*^	235,85^*^	367,92^*^	515,60^*^
	**Max**	1611,26	2797,68	3681,43	1347,81	956,54	1553,69	2083,40

LC subjects without neurological manifestations were compared with those reporting at least one neurological symptom. Data are expressed as minimum (Min), median (Median), and maximum (Max) values. Statistical differences between groups were evaluated using the non-parametric Kruskal–Wallis test. Asterisks indicate statistically significant differences between LC individuals with and without neurological symptoms (*p* < 0.05).

**Table 13:** Principal component analysis (PCA) of extracellular vesicle (EV) subpopulations, circulating cytokines, biochemical parameters and inflammation indices in long COVID (LC) individuals stratified according to the absence or presence of neurological symptoms.

LC individuals	NO_Neurologic symptomps				Neurologic symptomps					
		Components			Components					
		1	2	3	1	2	3	4	5	6
EVs/μL plasma	HLA-DR+CD169+	,989	,143	,032	,996	,038	,018	,057	,053	,001
	HLA-DR+	,994	,104	-,037	,996	,059	,040	,043	,038	,009
	CD169+	,992	,103	-,072	,998	,052	,001	,030	,024	,009
	HLA-DR+CD169+ (240_500 nm)	,975	,202	,088	,997	,014	,034	,051	,033	,014
	HLA-DR+ (240_500 nm)	,979	,194	,062	,998	,038	,013	,030	,025	,008
	CD169+ (240_500 nm)	,981	,194	,006	,999	,030	,017	,021	,016	,001
	HLA-DR+CD169+ (100_200 nm)	-,597	,301	,744	,367	-007	,494	,751	,095	,217
	HLA-DR+ (100_200 nm)	-,898	,383	,216	,162	,034	,351	,850	,173	,312
	CD169+ (100_200 nm)	-,897	,340	,283	,231	,014	,432	,806	,250	,218
	HLA-DR+CD169+ (500 nm)	,995	,101	,002	,991	,058	,016	,094	,077	,018
	HLA-DR+ (500 nm)	,991	,023	-,131	,991	,084	,071	,060	,051	,006
	CD169+ (500 nm)	,992	,024	-,121	,993	,074	,006	,074	,038	,024
Circulating Cytokines (pg/mL)	IL-1β	-,073	-,997	-,033	,373	,111	,116	,175	,896	,026
	IL-1RA	,651	,055	-,757	,854	,380	,236	,155	,013	,214
	CXCL10	,955	-,295	,015	,345	,014	,398	,108	,827	,166
	IL-6	-,403	-,914	,038	,525	,039	,059	,423	,401	,616
	IL-8	,147	-,989	,005	,881	,251	,088	,337	,167	,112
	TNFα	,890	-,404	-,213	,583	,007	,155	,067	,795	,007
	NFLs	,877	-,310	-,368	,320	,883	,192	,164	,005	,231
	GMCSF	-,428	,576	-,697	,005	,217	,056	,935	,110	,253
	CCL2	-,598	-,465	-,653	,296	,201	,647	,635	,206	,084
	Endothelin1	,850	,318	-,420	,507	,559	,036	,440	,048	,482
	ST2	,017	,443	,896	,919	,073	,091	,213	,109	,290
	TNFR1	,402	,440	,803	,616	,669	,067	,215	,126	,326
	IL-10	-,307	-,930	,202	,537	,778	,318	,040	,043	,034
	IL17A	,695	-,694	-,188	,110	,968	,187	,038	,001	,120
	IL-18	-,590	-,806	-,044	,759	,576	,209	,101	,043	,191
	ICAM	-,074	,954	,292	,615	,606	,351	,332	,145	,022
	VCAM	,749	,604	,273	,136	,954	,176	,096	,018	,174
Biochemical parameters and inflammation indices	Platelets	-,457	-,091	,885	,223	,341	,251	,112	,165	,855
	white blood cells	-,116	-,564	,817	,566	,164	,145	,352	,104	,705
	PT %	-,844	,024	-,536	,557	,632	,032	,347	400	,096
	PT-INR	,827	,022	,562	,547	,669	,043	,348	,345	,107
	PT sec	,862	-,085	,500	,598	,614	,155	,300	,387	,043
	aPTT ratio	-,514	,515	,686	,056	,328	,376	,406	,699	,308
	aPTT sec	,948	-,111	-,298	,212	,804	,088	,417	,181	,308
	Fibrinogen	,740	-,670	,063	,503	,396	,590	,264	,280	,307
	AST	-,119	,983	,136	,298	,564	,397	,298	,574	,133
	ALT	-,169	,964	-,207	,740	,525	,081	,149	,320	,214
	CRP	,766	,586	,263	,009	,063	,969	,238	,001	,010
	D-Dimer	,958	,242	-,151	,231	,954	,142	,098	,085	,010
	SII	-,157	-,064	,986	,438	,133	,819	,151	,162	,266
	NLR	,164	-,202	,966	,428	-,204	,831	,203	,184	,104
	PLR	-,173	,178	,969	,110	,014	,740	,176	,203	,606

The table reports component loadings for each variable across the extracted principal components in LC individuals without neurological symptoms and in those with neurological symptoms. EVs are expressed as number per µL of plasma and are further classified according to surface markers (HLA-DR and CD169) and size ranges. PCA was performed on standardized variables using a correlation matrix to identify distinct clustering patterns associated with neurological involvement in LC.

## 4 Discussion

In this study, we extended our previous findings by analyzing the expression patterns of CD169 and HLA-DR across distinct leukocyte subsets as well as in plasma-derived extracellular vesicles in patients with acute SARS-CoV-2 infection, individuals with long COVID, and healthy donors. We aimed to delineate the specific myeloid activation that characterizes both the acute and post-acute phases of SARS-CoV-2 infection, with particular attention to the correlation between cellular and extracellular vesicles. We first analyzed the ratio of CD169 MFI in HLA-DR^+^ monocytes vs. HLA-DR^+^ lymphocytes (CD169 RMFI). As we have previously described (Minutolo et al., 2021; Fanelli et al., 2023), CD169 RMFI was found significantly elevated in COV patients compared to the LC and HD groups, confirming its value as a marker of early viral infection. This was accompanied by increased CD169 expression in total leukocytes, especially monocytes, consistent with other reports showing CD169 upregulation in acute SARS-CoV-2 infection (Bedin et al., 2021; Bourgoin et al., 2021; Doehn et al., 2021). CD169 (Siglec-1) is known to bind gangliosides (GM1, GM3) on SARS-CoV-2 membranes, facilitating virus–cell interactions and trans-infection of ACE2^+^ cells (Zhou et al., 2020; Perez-Zsolt et al., 2021). Moreover, CD169 enables ACE2-independent entry into macrophages, where it promotes partial viral replication restricted at the post-entry stage (Jalloh et al., 2022). In LC individuals, despite the absence of active infection, CD169 expression remained moderately elevated, particularly on monocytes and to a lesser extent on granulocytes, suggesting persistent immune activation and innate immune dysregulation. Notably, CD169^+^ monocytes have been associated with poor lung function and elevated cytokine levels in LC (Park et al., 2023), supporting their potential role in driving chronic inflammation. Although HLA-DR expression in total leukocytes was not different among groups, subset-specific analysis revealed significant alterations. In monocytes, both COV and LC individuals displayed reduced HLA-DR expression compared to HD, a finding consistent with previous studies linking low monocytic HLA-DR (mHLA-DR) to severe disease and prolonged hospitalization (Gatti et al., 2020; Qin et al., 2021; Fanelli et al., 2023). This downregulation may be mediated by the cytokine storm, particularly IL-6 and IL-10, as mHLA-DR expression negatively correlates with their circulating levels and is partially restored following IL-6 blockade with tocilizumab (Giamarellos-Bourboulis et al., 2020). Interestingly, while monocytes showed impaired antigen presentation capacity, HLA-DR expression on lymphocytes was elevated in both COV and LC compared to HD. HLA-DR is a known activation marker on T cells and has been observed to be modulated in various inflammatory and infectious settings, including HIV and SARS-CoV-2 (Xia et al., 2018; Reddy et al., 2004). This suggests that T-cell activation persists beyond the acute phase and may contribute to ongoing immune dysregulation in LC.

A crucial aspect of our study was the characterization of HLA-DR^+^CD169^+^ double-positive leukocytes and the corresponding expression in EVs. At the cellular level, we confirmed that the presence of monocytes was able to discriminate both COV and LC from HD. Moreover, despite the lower frequency, the persistence of HLA-DR^+^CD169^+^ monocytes in LC suggests the presence of residual viral molecules or of a chronic stimulation. Several studies have demonstrated that circulating extracellular vesicles can display viral antigens, supporting the notion of prolonged immune activation in LC (Barberis et al., 2021; Campello et al., 2022). Notably, herein, we have quantified the plasmatic Spike S1-positive EVs by flow cytometry, demonstrating high levels and persistence of Spike antigens carried by EVs in LC compared to HD.

In COVID-19 patients, the correlation analysis revealed that the number of HLA-DR^+^CD169^+^ EVs was strongly associated with the frequency of HLA-DR^+^CD169^+^ monocytes, as well as with CD169 RMFI values. This suggests that EV release mirrors cellular activation and could serve as circulating indicators of immune cell status. Notably, the number of HLA-DR^+^CD169^+^ EVs/µL of plasma increased proportionally with monocyte activation, despite an inverse trend in percentage values. Notably, the percentages of plasmatic HLA-DR^+^CD169^+^ EVs in patients ranged between 9.35 to 14.80 of the total circulating EVs, highlighting their high representation. Similarly, LC showed higher circulating HLA-DR^+^CD169^+^ EVs in percentage and events/µL compared to HD. Our study characterized for the first time through flow cytometry, in both percentage and absolute numbers, that HLA-DR and CD169 are present on plasmatic EVs and are dynamically expressed in healthy donors, COVID patients, and LC individuals. Particularly, we demonstrated that the plasmatic HLA-DR^+^CD169^+^ EVs characterize both COV and LC as potential novel biomarkers. Nonetheless, we acknowledge that the HLA-DRB1 genotype could modulate the amplitude or setpoint of HLA-DR expression and might help explain some of the interindividual variability we observed. Our cohort size and study design did not allow us to perform high-resolution HLA typing or to analyze allele-level associations, which is an important limitation. Future studies with larger cohorts will incorporate HLA-DRB1 genotyping (or imputation) to dissect how genetic background interacts with disease-driven immune dysregulation.

Biochemical parameters correlated distinctly with HLA-DR^+^ and CD169^+^ EV subsets in the COV and LC groups. Only a few inverse correlations were observed in COV, while several positive correlations were found in LC, particularly with parameters associated with coagulation (PT%, PT-INR) and with inflammatory (CRP, SII, NLR, and PLR) and hepatic functions (LDH, ALT). These associations may reflect persistent subclinical organ dysfunction and altered immune-EV dynamics. Systemic inflammation indices (SII, NLR, and PLR) were consistently associated with HLA-DR^+^CD169^-^ EVs, sustaining a low-grade inflammatory state in LC. The persistence of inflammation in LC is confirmed by the high concentration of circulating cytokines. It should be noted that their concentration is directly correlated with HLA-DR^+^ and CD169^+^ leukocytes. It is important to emphasize that the correlations observed between specific EV subsets and biochemical or inflammatory markers represent associations rather than mechanistic relationships. Our cross-sectional design does not allow causal inference; therefore, the detected links between EV phenotypes and coagulation, hepatic, or inflammatory parameters should be interpreted as reflective of concurrent immunological states rather than direct biological effects. These associations, nonetheless, highlight clinically relevant patterns of co-variation that warrant further mechanistic investigation.

The present study reveals distinct patterns of correlation between circulating cytokines and specific EV subtypes in patients with acute COV and those with LC, suggesting that EV phenotypes are dynamically shaped by the evolving immune microenvironment during the acute and post-infection phase. Indeed, in COV, we observed that pro-inflammatory cytokines such as IL-8 were positively associated with HLA-DR^+^CD169^+^ EVs and large HLA-DR^+^ EVs, supporting the concept that early immune activation leads to the expansion of myeloid- and antigen-presenting cell (APC)-derived vesicles. These results are in line with previous studies, reporting that IL-8 is highly expressed during acute SARS-CoV-2 infection and is involved in neutrophil recruitment and local tissue inflammation (Del Valle et al., 2020). Conversely, TNF-α and IL-10 were negatively correlated with small and large HLA-DR^+^ EVs, respectively. Interestingly, the vasoactive molecule endothelin-1 exhibited a size-dependent dual effect: it was positively correlated with mid-sized CD169^+^ EVs and negatively with small HLA-DR^+^ EVs. Endothelin-1 is a known mediator of endothelial dysfunction, and its correlation with CD169^+^ EVs suggests a link between vascular stress and myeloid cell activation (Rajendran et al., 2014). Moreover, the inverse association of CCL2 with multiple HLA-DR^+^ and CD169^+^ EV subsets in COV may reflect monocyte exhaustion or impaired APC function under chronic inflammatory stress, a feature commonly reported in severe COVID-19 (Kvedaraite et al., 2021). Notably, IL-2, CXCL10, and CCL2 cytokines involved in T-cell and monocyte function were significantly associated with CD169^+^ and HLA-DR^+^CD169^+^ EVs in LC. These patterns imply that Th1-driven immune signaling and monocyte activation continue to influence EV dynamics beyond the acute phase, potentially contributing to sustained immune activation in LC. In particular, CXCL10, a chemokine upregulated by IFN-γ, was associated with HLA-DR^-^CD169^+^ and HLA-DR^+^CD169^+^ EVs, sustaining the persistent interferon signatures in long COVID (Su et al., 2022). Furthermore, endothelin-1 and tissue remodeling (ST2, IL-4) were also positively correlated with distinct EV subsets in LC. These findings support the hypothesis that vascular injury and tissue repair processes continue to shape the EV landscape in the post-acute phase (Arnold et al., 2021).

Notably, the analysis of EV cell-derived compartments highlighted that HD samples displayed a balanced EV profile characterized by stable monocyte- and platelet-derived EVs and low immune activation markers. In contrast, COV patients showed a profound remodeling of EV composition, marked by interferon-activated monocyte EVs (CD169^+^), suppression of lymphocyte-derived EVs, and increased NK- and platelet-activated EVs. LC subjects exhibited an intermediate but distinct EV signature, characterized by persistent CD169^+^ and NK-derived EVs and re-emergence of T-cell-associated EVs, supporting the presence of chronic immune dysregulation rather than complete immune resolution. Notably, HD samples displayed low levels of Spike-positive and inflammatory EVs, consistent with immune homeostasis. In contrast, COV patients exhibited a marked enrichment of EVs carrying viral antigens and interferon-related chemokines, reflecting active infection and systemic immune activation. LC subjects showed an intermediate but distinct EV profile, characterized by persistent Spike RBD and IL-6/TNF-α-associated EVs, supporting the concept of ongoing antigenic stimulation and chronic low-grade inflammation after SARS-CoV-2 infection. Furthermore, the size distribution obtained using AFM showed how the sizes of EVs are reduced comparing HD and COV, while the LC appears to show diameter distributions, which are similar to those measured in the HD cases (**Supplementary Material 1**), underlining the importance of the characterization of EV derivation and cargo.

In addition, a first stratification comparing LC patients without any symptoms (9/25) to those reporting at least one symptom (16/25) revealed statistically significant differences in neutrophil and lymphocyte percentages, as well as in the inflammatory indices SII and NLR, together with altered potassium levels. These findings suggest that even mildly symptomatic LC individuals display measurable shifts in systemic inflammatory balance, consistent with previous evidence of persistent low-grade inflammation and immune activation in LC cohorts (Phetsouphanh et al., 2022; Schultheiß et al., 2022). When stratifying patients according to the presence of systemic symptoms, significant differences emerged in IL-1β, IL-1RA, TNF, and IL-10 levels, along with platelet counts. This cytokine pattern aligns with reports describing dysregulated innate immune responses in LC, particularly involving the IL-1 and TNF pathways (Hanke et al., 2022; Fogarty et al., 2022). The concomitant increase in IL-10 may reflect compensatory anti-inflammatory signaling, a feature previously documented in chronic post-viral syndromes (Chioh et al., 2021). No significant differences were observed in CD169^+^ and HLA-DR^+^ EV populations across these comparisons, suggesting that EV-mediated immune signaling does not globally distinguish symptomatic from asymptomatic LC profiles. This is consistent with the heterogeneity in EV signatures reported in LC studies and the lack of broad consensus on EV markers for LC stratification (Squitti et al., 2023). However, a distinct pattern emerged when focusing on neurological symptoms. Patients reporting neurological manifestations (14/25) showed significantly decreased CRP and potassium levels, and, notably, a significant reduction in the concentration (events/µL) of CD169^+^/HLA-DR^+^ EVs, particularly within the >500- and 240–500-nm size ranges. Since both CD169 and HLA-DR are associated with monocyte/macrophage activation and antigen presentation, their decreased EV output may indicate impaired myeloid signaling, echoing reports of dysfunctional monocyte phenotypes in LC with neurological involvement (Theoharides et al., 2023; Ong et al., 2022). These findings support the hypothesis that neuro-LC may involve specific alterations in neuroimmune communication, potentially mediated by EV-dependent pathways (Rauch et al., 2022). Overall, these stratified analyses indicate that different LC symptom clusters associate with distinct immune signatures, with neurological symptoms showing the most specific EV-related phenotype. This observation strengthens current models proposing that altered myeloid-derived vesicle trafficking and antigen presentation may contribute to LC neuropathophysiology (Proal and VanElzakker, 2021; Swank et al., 2022). By PCA analysis, the expansion from three components in no-neuro patients to six in those with neurological symptoms indicates a marked disruption of the global immunological organization in the neuro subgroup. While no-neuro subjects showed coordinated axes integrating monocyte activation, cytokine signaling, and hematologic/coagulation responses, neuro patients exhibited a fragmented immune structure, suggesting reduced cross-talk between innate, endothelial, and systemic inflammatory pathways. The exclusive emergence of an IL-17A/VCAM/NFL component strongly supports a neuroinflammatory process. IL-17A is known to promote endothelial activation and blood–brain barrier (BBB) permeability (Korn et al., 2009), while its association with VCAM suggests enhanced leukocyte adhesion and potential BBB dysfunction (Daneman and Prat, 2015). The parallel loading of NFLs—markers of axonal injury—links these immune alterations to measurable neuronal damage, a pattern consistent with recent observations in long COVID cohorts with neurological involvement (Lee et al., 2022). The segregation of GM-CSF and CCL2 into an independent component indicates altered myeloid–cell recruitment dynamics. Both cytokines drive monocyte trafficking and differentiation (Shi and Pamer, 2011), and their dissociation from monocyte activation markers may reflect a decoupled, dysregulated myeloid response, as described in chronic post-viral inflammation (Schultze and Aschenbrenner, 2021). Similarly, the separation of IL-1β, TNF-α, and CXCL10 from the acute-phase axis suggests that neuro LC patients maintain cytokine activation that is not synchronized with systemic inflammatory markers—unlike the more integrated response observed in no-neuro individuals. CXCL10, in particular, has been implicated in persistent neuroimmune activation and cognitive symptoms after viral infections (Heming et al., 2022). Finally, the formation of an isolated hematologic component (platelets, WBC) further supports a dissociation between systemic and cellular inflammatory modules, which may contribute to microvascular and endothelial perturbations linked to neurological long COVID phenotypes (Pretorius et al., 2022). Overall, the immune landscape of neuro LC patients appears fragmented and with specific activation of Th17-endothelial pathways and evidence of neuronal injury, potentially reflecting BBB impairment and neuroimmune dysregulation.

Taken together, these data highlight a fundamental change in the regulation of EVs, which are diverse in size and phenotype in LC compared to COV. It will be needed to increase the number of patients and donors for validating the obtained data. Also, a deeper characterization of the extracellular vesicle content would be useful to clarify their role in intercellular communication at both the physiological and pathological levels.

Our findings support a model in which EV profiling stratified by surface markers and size can provide insights into ongoing immunopathological processes in both acute and chronic COVID-19. Given the pleiotropic functions of EVs in cell–cell communication, antigen presentation, and inflammation, further exploration of their cargo and origin is needed to better understand their functional role in disease progression and recovery. Overall, our findings point to a strict interplay between immune cell activation, extracellular vesicle release, and systemic pathophysiology in both acute and post-acute COVID-19. The cellular and extracellular expression of CD169 and HLA-DR, along with their associations with cytokines and biochemical markers, underscore their utility as integrated biomarkers of immune dysregulation. These insights support the hypothesis that long COVID represents a distinct immunopathological state, characterized by persistent immune activation, metabolic stress, and organ-specific dysfunction, potentially sustained by EV-mediated signaling.

These findings propose EVs as sensitive indicators of both acute and persistent immune perturbations, bridging viral antigen persistence with inflammatory signaling in long COVID. The analysis of these markers on a large number of samples could consistently define specific profiles associated with different clinical manifestations. Moreover, in a complex clinical context of heterogeneous manifestations such as long COVID, the identification of the co-expression of HLA-DR^+^ and CD169^+^, both at the cellular level in blood and in plasma extracellular vesicles, may serve as novel potential biomarkers.

## Data Availability

The raw data supporting the conclusions of this article will be made available by the authors, without undue reservation.
