# Supplementary material for: CD169+ and HLA-DR+ extracellular vesicles are highly represented in human plasma and dynamically expressed in SARS-CoV-2 infection and long COVID-associated sequelae
Source: Front Cell Infect Microbiol. 2026 Feb 11;16:1686186. doi: 10.3389/fcimb.2026.1686186 (PMC12932623; doi:10.3389/fcimb.2026.1686186)

Supplementary Material

# EV size determination using AFM Supplementary Figure 1A. Microvesicles diameter distribution of HD (left panel), COVID-19 patients (COV) (centre panel) and long COVID (LC) (right panel). All curves were fitted with gaussian curves to determine the presence of populations of EVs with different diameters.

#
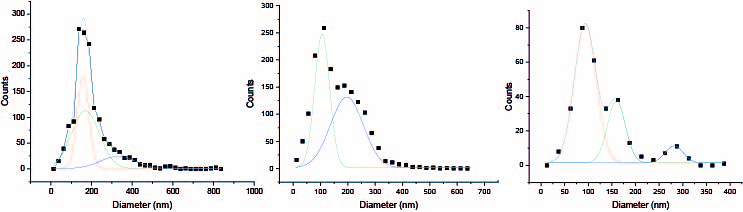
The size distribution evidences the presence of EVs of different diameters. The data were plotted and fitted using gaussian curves. In the HD, the data show three distinct populations of sizes of 159 ± 39,20 nm; 170 ± 72,9 nm; 329 ± 53,0 nm. In COV only two populations can be seen, centered at 106 ± 28,44 nm and 196 ± 46,6 nm. In LC the distribution of EVs was characterized by three populations with lateral width of 93,7 ± 20,81 nm; 159 ± 61,78 nm; 282 ± 54,69 nm.

Overall, differences between EVs from different groups can be seen. HD and LC cases show diameters which are peaked around 160 nm and 300 nm, with a more distinct separation between the two low-diameter populations in the LC cases. On the other hand, the COV cases show a different size behaviour, where only two populations can be seen, with diameters which are smaller than the corresponding ones measured in HD and LC.

# Supplementary Figure 1B. Typical AFM morphology of EVs from HD, COV and LC. All images are 5x5 micron.


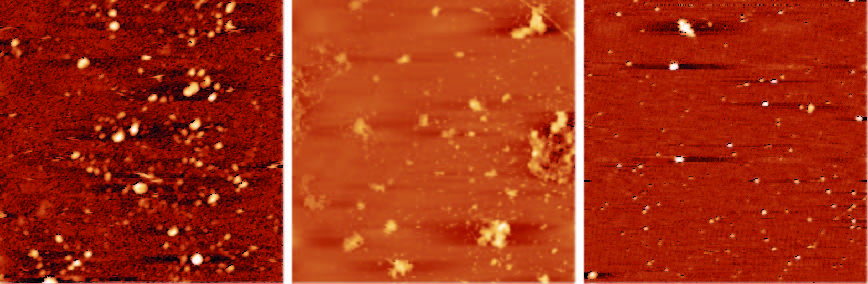


# Supplementary figure 2. Gating strategy of HLA-DR+, CD169+, HLA-DR+CD169+ leukocytes subpopulations through flow cytometry.


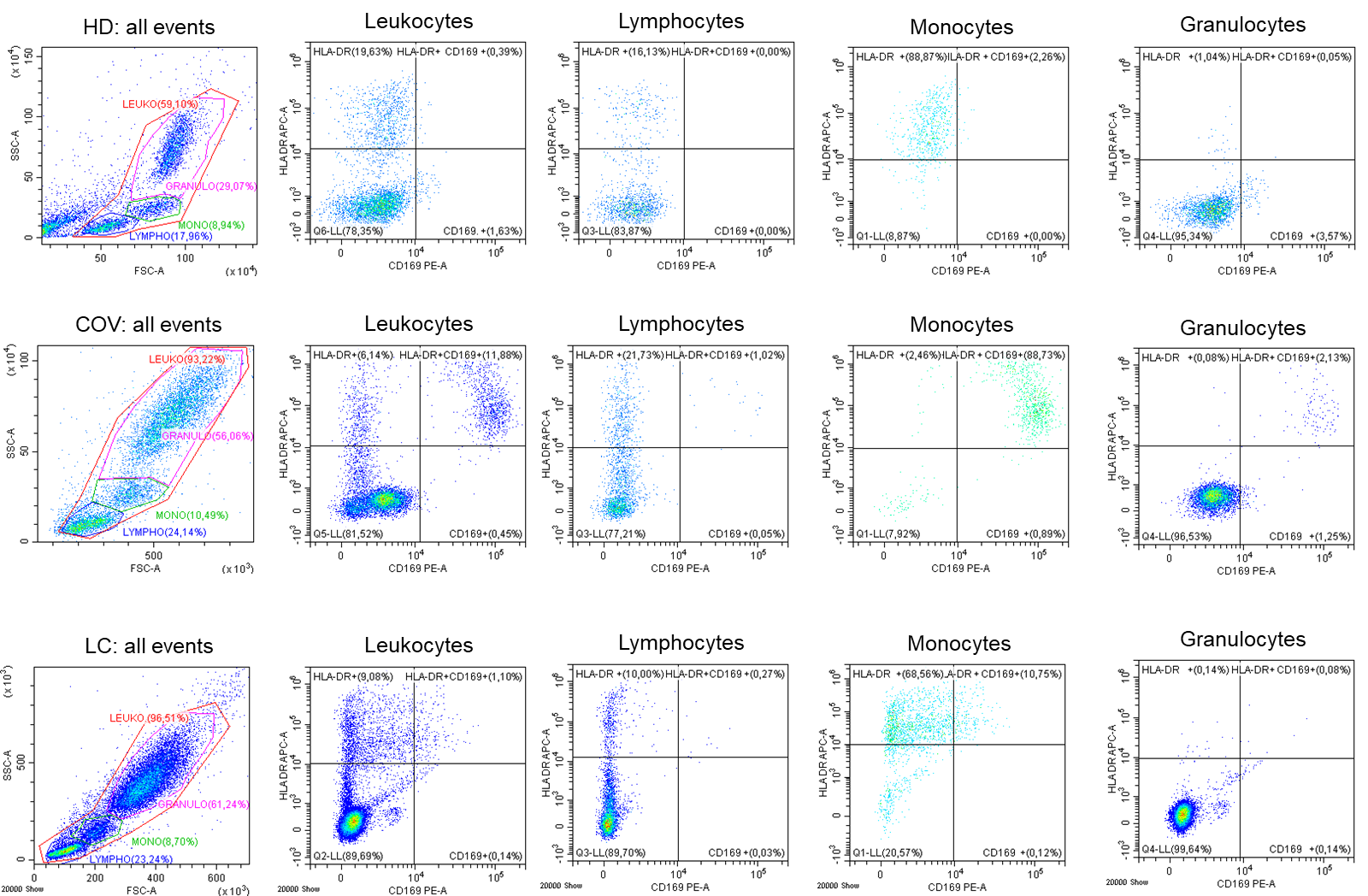


# Figure 3. Gating strategy of HLA-DR+, CD169+, HLA-DR+CD169+ EVs subpopulations through flow cytometry.


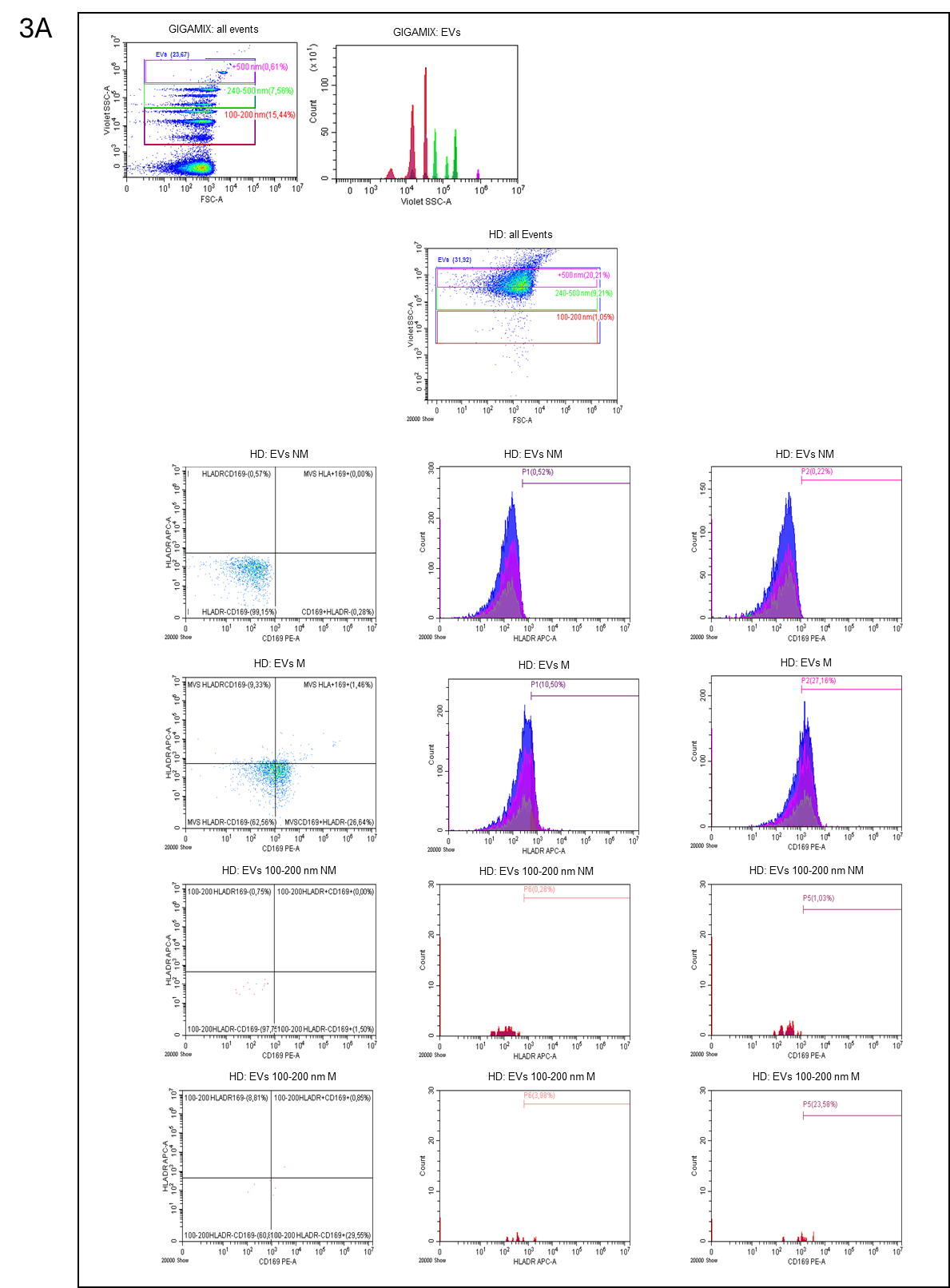


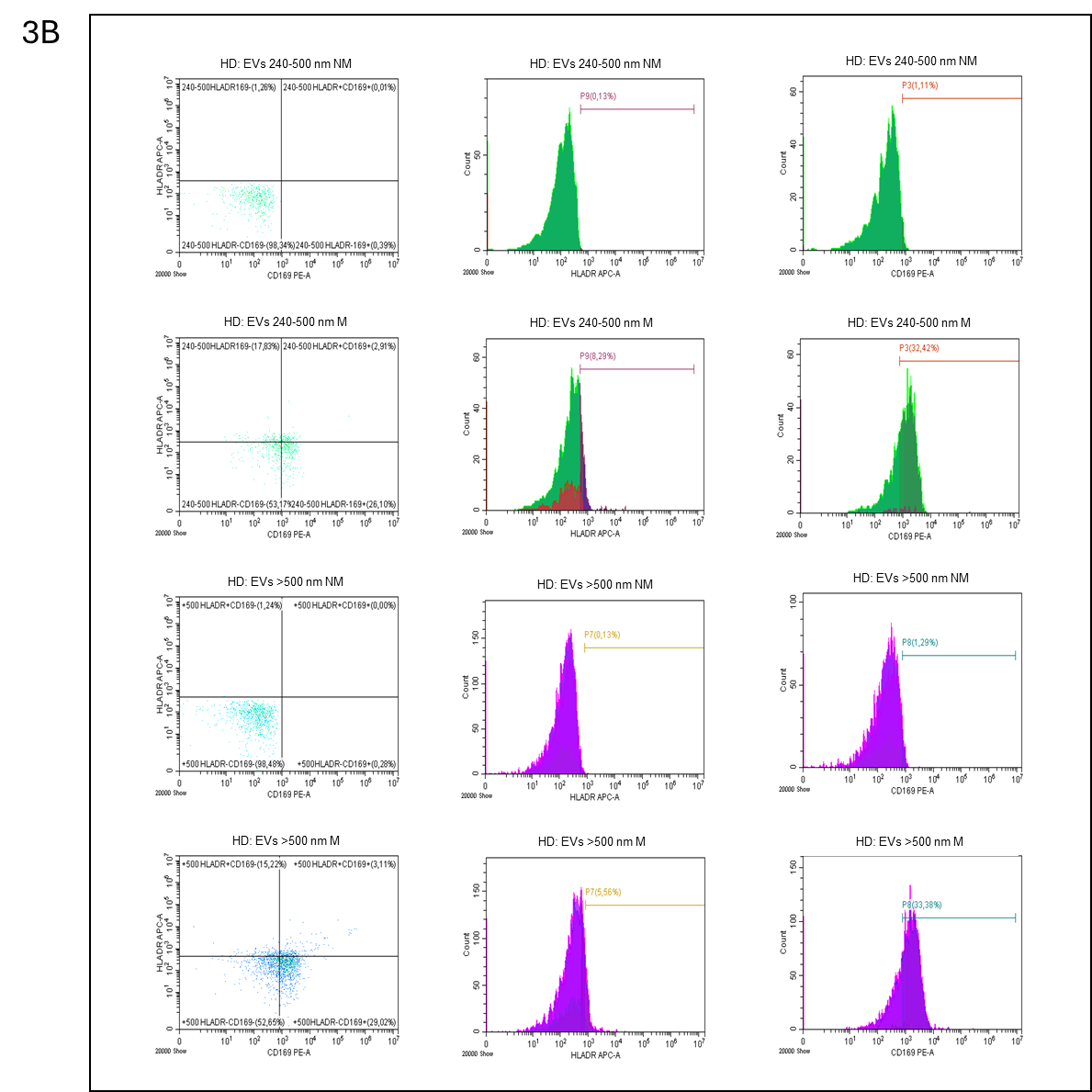


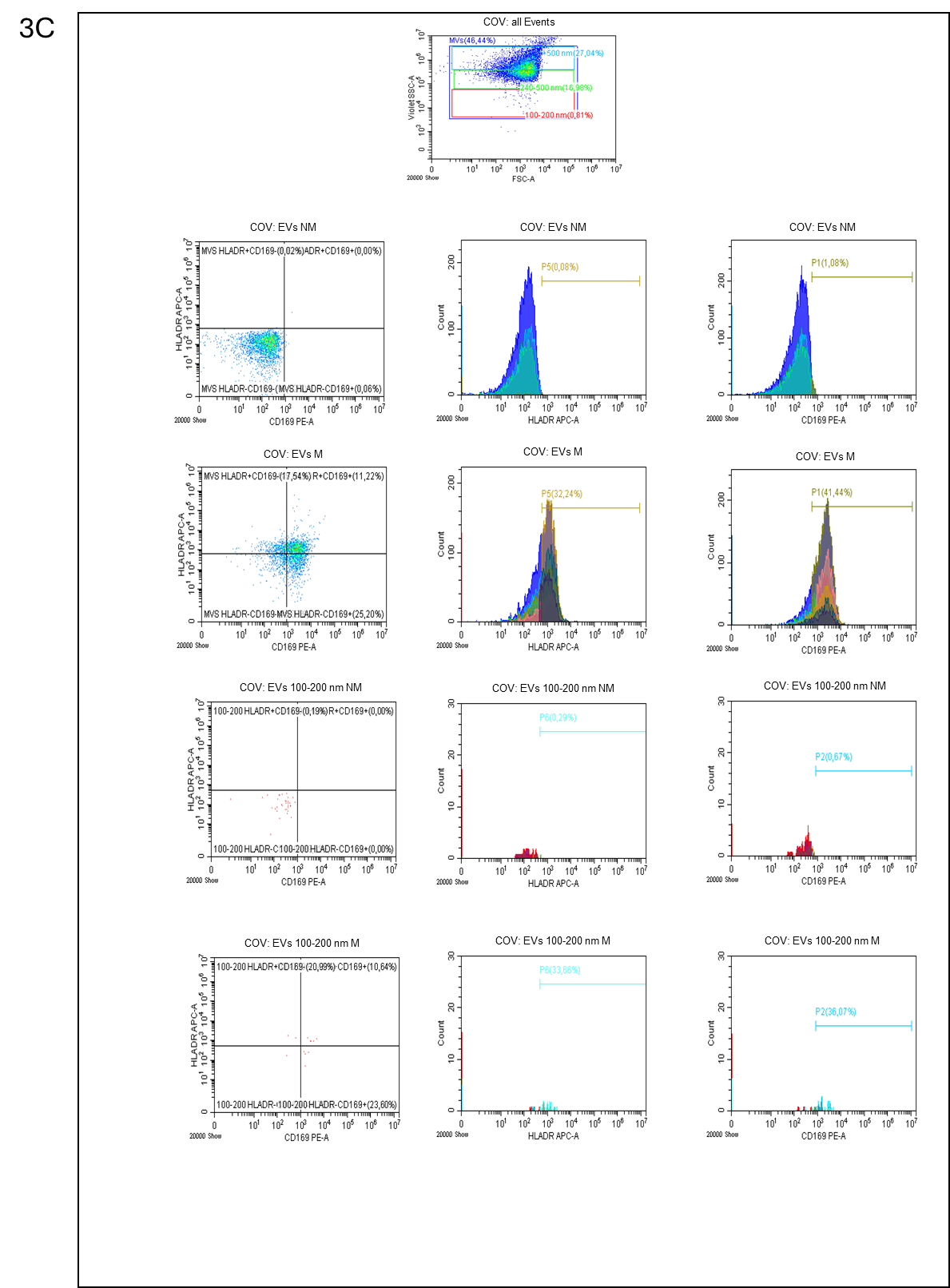


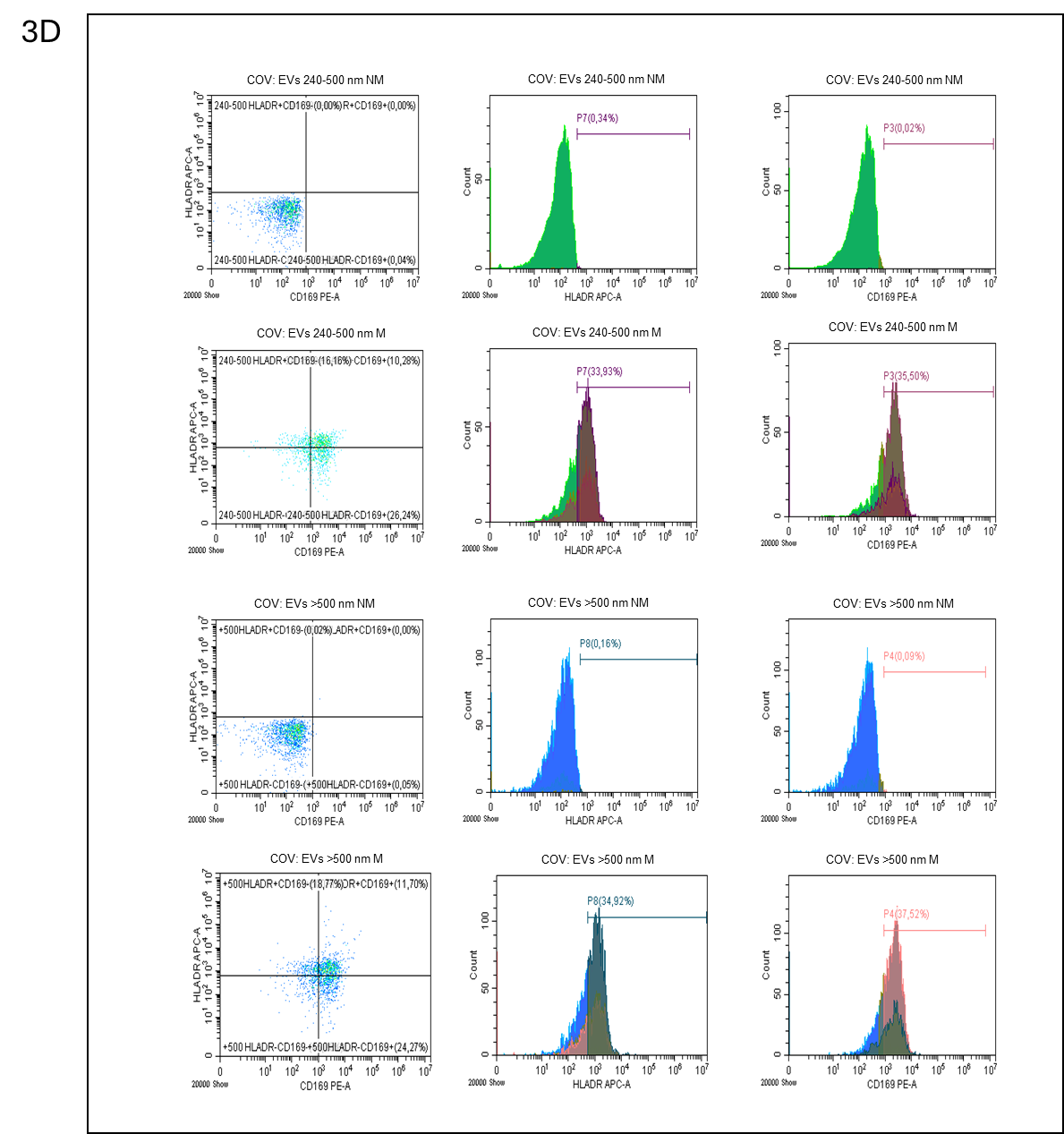


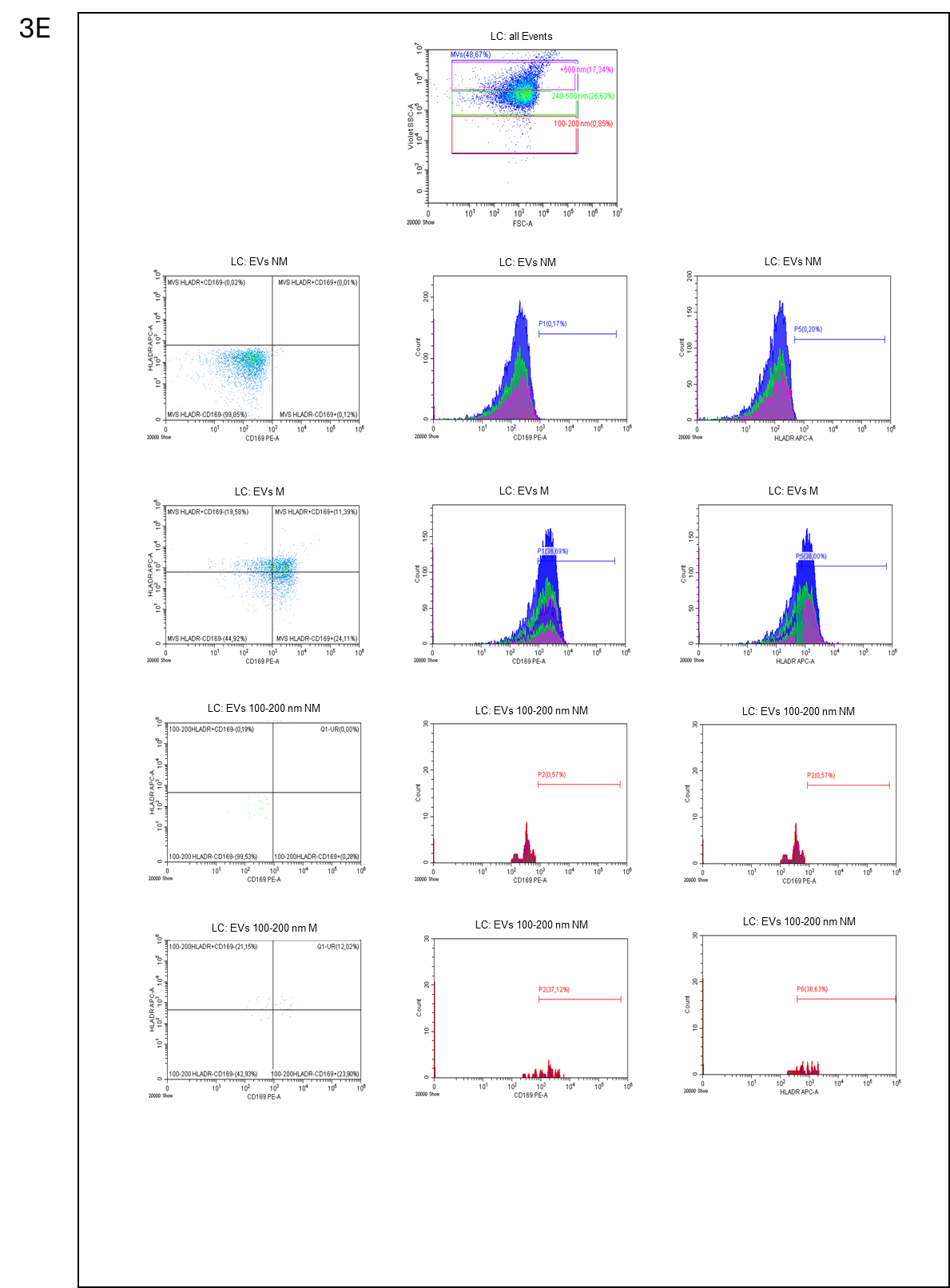


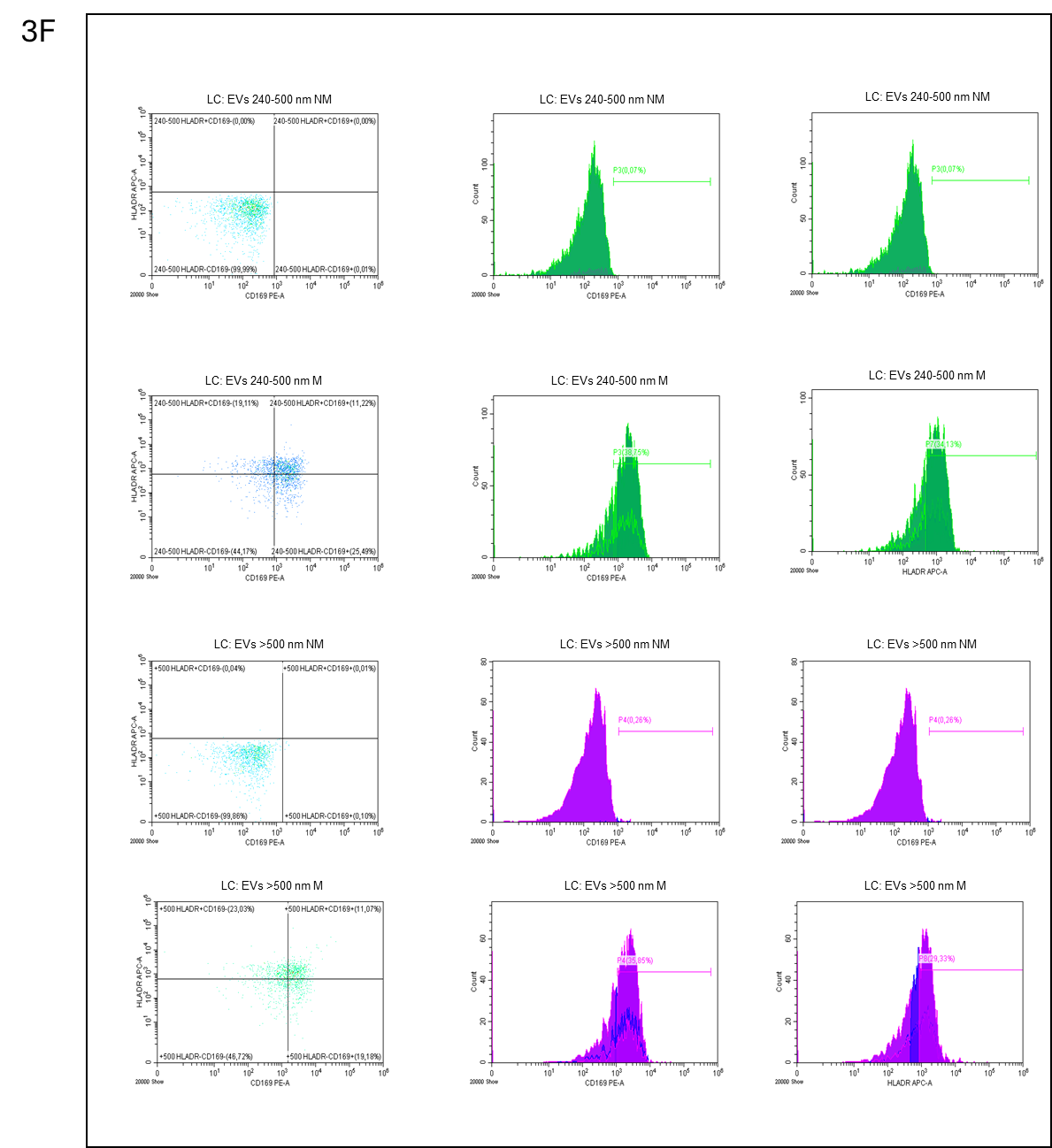


# Supplementary Figure 4. Evaluation of EVs and PBS filtered

#
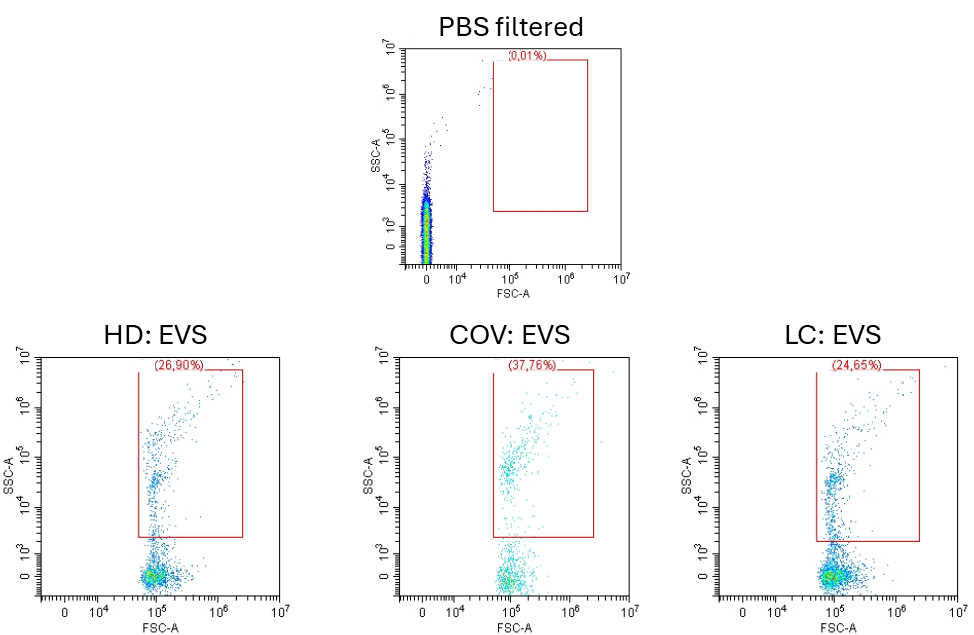

Supplement: Supplementary file 1 [file Table1.docx]
